# Effects of water, sanitation, handwashing and nutritional interventions on soil-transmitted helminth infections in young children: A cluster-randomized controlled trial in rural Bangladesh

**DOI:** 10.1371/journal.pntd.0007323

**Published:** 2019-05-03

**Authors:** Ayse Ercumen, Jade Benjamin-Chung, Benjamin F. Arnold, Audrie Lin, Alan E. Hubbard, Christine Stewart, Zahidur Rahman, Sarker Masud Parvez, Leanne Unicomb, Mahbubur Rahman, Rashidul Haque, John M. Colford, Stephen P. Luby

**Affiliations:** 1 Department of Forestry and Environmental Resources, North Carolina State University, Raleigh, NC, United States of America; 2 School of Public Health, University of California Berkeley, Berkeley, CA, United States of America; 3 Department of Nutrition, University of California, Davis, CA, United States of America; 4 Infectious Disease Division, International Centre for Diarrhoeal Disease Research, Bangladesh, Dhaka, Bangladesh; 5 Infectious Diseases & Geographic Medicine, Stanford University, Stanford, CA, United States of America; University of New South Wales Faculty of Medicine, AUSTRALIA

## Abstract

**Background:**

Soil transmitted helminths (STH) infect >1.5 billion people. Mass drug administration (MDA) effectively reduces infection; however, there is evidence for rapid reinfection and risk of potential drug resistance. We conducted a randomized controlled trial in Bangladesh (WASH Benefits, NCT01590095) to assess whether water, sanitation, hygiene and nutrition interventions, alone and combined, reduce STH in a setting with ongoing MDA.

**Methodology/Principal findings:**

In 2012–2013, we randomized 720 clusters of 5551 pregnant women into water treatment, sanitation, handwashing, combined water+sanitation+handwashing (WSH), nutrition, nutrition+WSH (N+WSH) or control arms. In 2015–2016, we enrolled 7795 children, aged 2–12 years, of 4102 available women for STH follow-up and collected stool from 7187. We enumerated STH infections with Kato-Katz. We estimated intention-to-treat intervention effects on infection prevalence and intensity. Participants and field staff were not blinded; laboratory technicians and data analysts were blinded.

Prevalence among controls was 36.8% for *A*. *lumbricoides*, 9.2% for hookworm and 7.5% for *T*. *trichiura*. Most infections were low-intensity. Compared to controls, the water intervention reduced hookworm by 31% (prevalence ratio [PR] = 0.69 (0.50,0.95), prevalence difference [PD] = -2.83 (-5.16,-0.50)) but did not affect other STH. Sanitation improvements reduced *T*. *trichiura* by 29% (PR = 0.71 (0.52,0.98), PD = -2.17 (-4.03,-0.38)), had a similar borderline effect on hookworm and no effect on *A*. *lumbricoides*. Handwashing and nutrition interventions did not reduce any STH. WSH and N+WSH reduced hookworm prevalence by 29–33% (WSH: PR = 0.71 (0.52,0.99), PD = -2.63 (-4.95,-0.31); N+WSH: PR = 0.67 (0.50,0.91), PD = -3.00 (-5.14,-0.85)) and marginally reduced *A*. *lumbricoides*. Effects on infection intensity were similar.

**Conclusions/Significance:**

In a low-intensity infection setting with MDA, we found modest but sustained hookworm reduction from water treatment and combined WSH interventions. Impacts were more pronounced on STH species with short vs. long-term environmental survival. Our findings suggest possible waterborne transmission for hookworm. Water treatment and sanitation improvements can augment MDA to interrupt STH transmission.

**Trial registration:**

NCT01590095.

## Author summary

Soil-transmitted helminths (STH) are associated with a large disease burden worldwide. Mass administration of deworming drugs for preventive chemotherapy is the cornerstone of global strategy for STH control, but treated individuals often rapidly become reinfected, and there is also concern about emerging drug resistance. Interventions to treat drinking water, wash hands at critical times and isolate human feces from the environment through improved sanitation could reduce STH transmission by reducing the spread of ova from the feces of infected individuals into the environment and subsequently to new hosts. Nutrition improvements could reduce host susceptibility to infection. Existing evidence on the effect of these interventions on STH is scarce. In a setting with ongoing mass drug administration, we assessed the effect of individual and combined water, sanitation, handwashing and nutrition interventions on STH infection in children. Approximately 2.5 years after the initiation of interventions, we found reductions in STH infection from water treatment and sanitation interventions; there was no reduction from the handwashing and nutrition interventions. While the reductions were modest in magnitude compared to cure rates achieved by deworming drugs, they indicated sustained reduction in environmental transmission. The reductions were more pronounced for STH species that do not have long-term environmental reservoirs. These findings suggest that water treatment and sanitation interventions can augment mass drug administration programs in striving toward elimination of STH.

## Introduction

Over 1.5 billion people globally are infected with soil transmitted helminths (STH), specifically 819 million with *Ascaris lumbricoides* (roundworm), 465 million with *Trichuris trichiura* (whipworm), and 439 million with *Necator americanus* and *Ancylostoma duodenale* (hookworms) [1,2]. Deworming with mass drug administration (MDA) for preventive chemotherapy is the cornerstone of global WHO policy for STH control and effectively reduces infection [3]. However, risk of drug resistance threatens the effectiveness of MDA programs given the wide-scale use, inadequate monitoring and limited number of effective anthelminthics, and frequent anthelminthic resistance in livestock [4]. Additionally, without environmental interventions to interrupt transmission, rapid reinfection is common; a systematic review demonstrated that prevalence reverts to 94% of pre-treatment levels for *A*. *lumbricoides*, 82% for *T*. *trichiura* and 57% for hookworm within 12 months post-treatment [5].

Water, sanitation and hygiene improvements could potentially complement MDA programs in reducing STH transmission [6]. Two systematic reviews found reduced STH infection associated with improved water, sanitation and hygiene conditions in observational studies [7,8]; however, there are few randomized assessments of the effect of water, sanitation and hygiene interventions on STH [6]. School-based hygiene education trials have had mixed effects on STH. A health education program in Chinese schools improved handwashing practices and reduced STH infection [9] while health hygiene education in schools in Peru reduced infections with *A*. *lumbricoides* but not *T*. *trichiura* and hookworm [10]. Handwashing with soap and fingernail clipping reduced parasite infections in children in a trial in Ethiopia [11]. Two trials in India found no STH reduction from sanitation improvements, potentially because they did not attain sufficiently high latrine usage [12,13]. It is also possible that persistent long-term environmental reservoirs of STH ova sustain infections given the prolonged survival of some STH species, such as *A*. *lumbricoides*, in soil [14]. While sanitation improvements should reduce immediate fecal input into the environment, their protective effect against STH infections may not be apparent until pre-existing ova in the environment are naturally inactivated [15].

Combined water, sanitation and hygiene improvements targeting multiple transmission routes might achieve a larger impact by complementing the primary barrier of sanitation with the secondary barriers of water treatment and handwashing [16,17]. School-based provision of combined water, sanitation and hygiene hardware reduced reinfection with *A*. *lumbricoides* but not other STH in a Kenyan trial [18]. A recent community-based trial of integrated water, sanitation and hygiene interventions in addition to deworming in Timor-Leste found no impact on STH infections compared to deworming alone [19].

The effect of nutrition on STH infections is also poorly understood. Impaired immune function from nutritional deficiencies could increase host susceptibility to STH infection or exacerbate infection severity while nutritional supplements could also increase infection severity as excess nutrients are available for pathogens [20,21]. A systematic review found mixed impacts of nutritional supplements on STH infection, concluding that the evidence is scarce and low-quality [20]. Nutrition interventions alongside water, sanitation and hygiene improvements could achieve synergistic benefits against STH infections.

We conducted a cluster-randomized trial (WASH Benefits, NCT01590095) in Bangladesh to assess the impact of individual and combined water, sanitation, handwashing (WSH) and nutrition interventions on child diarrhea and growth (primary and secondary outcomes) [22]. The trial found that all interventions except for the individual water intervention reduced reported diarrhea, and all interventions with a nutrition component improved linear growth [23]. Here, we report trial findings on STH infections (pre-specified tertiary outcomes) and test the hypotheses that (1) individual and combined WSH and nutrition interventions reduce STH infections, (2) combined WSH interventions reduce STH infections more than individual WSH interventions, and (3) combined nutrition and WSH interventions reduce STH infections more than nutrition or WSH interventions alone. This work provides a novel investigation of the effect of improved WSH and nutrition on STH infections in a population with ongoing MDA to inform policy dialogue on whether these can complement MDA programs.

## Methods

### Study setting

The trial was conducted in the Gazipur, Mymensingh, Tangail and Kishoreganj districts of central rural Bangladesh, which were selected because they had low groundwater arsenic and iron (to not interfere with the trial’s chlorine-based water intervention) and no other water, sanitation, hygiene or nutrition programs. The majority of households in the study area relied on untreated tubewell water for drinking, and while most households had access to on-site sanitation, it was common for latrines to drain into the environment, and animal feces were also commonly observed in the compounds [23,24].

Since 2008, the Bangladesh Ministry of Health has implemented a school-based MDA program that provides deworming to school-aged children while pre-school-aged children receive deworming through the Expanded Program on Immunization (EPI). The school-based MDA offers a single dose of mebendazole biannually while the EPI uses albendazole [25]. In a single dose, both drugs are effective for *A*. *lumbricoides* but have lower cure rates for *T*. *trichiura*; for hookworm, albendazole has a high cure rate while mebendazole has a modest cure rate [3,26]. A 2010 evaluation of the national MDA campaign in two districts (not included in the WASH Benefits trial) found that 63–73% of school-attending children, 11–14% of non-school-attending school-aged children and 60% of pre-school-aged children received deworming [25]. WASH Benefits activities were implemented independently from the MDA and EPI programs.

### Randomization and masking

The WASH Benefits trial enrolled pregnant women in their first or second trimester intending to stay in their village for 24 months post-enrollment, with the objective of following the birth cohort (“index children”) born to them. Field staff screened the study area for pregnant women and collected the global positioning system (GPS) coordinates of the compounds they lived in. Eight neighboring eligible women were grouped into clusters using their compounds’ GPS coordinates. Cluster dimensions were chosen such that one field worker could visit all participants in a cluster in one day. A minimum 1-km buffer was enforced between clusters to minimize spillovers of infections and/or intervention behaviors between study arms. Every eight adjacent clusters enrolled formed a geographic block. An off-site investigator (BFA) used a random number generator to block-randomize clusters into study arms, providing geographically pair-matched randomization. Participants and field staff were not blinded as interventions entailed distinct hardware; blinded technicians enumerated STH outcomes and blinded analysts (AE, JBC) independently replicated data management and analysis. Details of the study design have been previously described [22]. The study protocol, pre-specified analysis plan, and a CONSORT checklist of trial procedures have been provided (S1–S3 Text).

### Interventions

**Fig 1:**
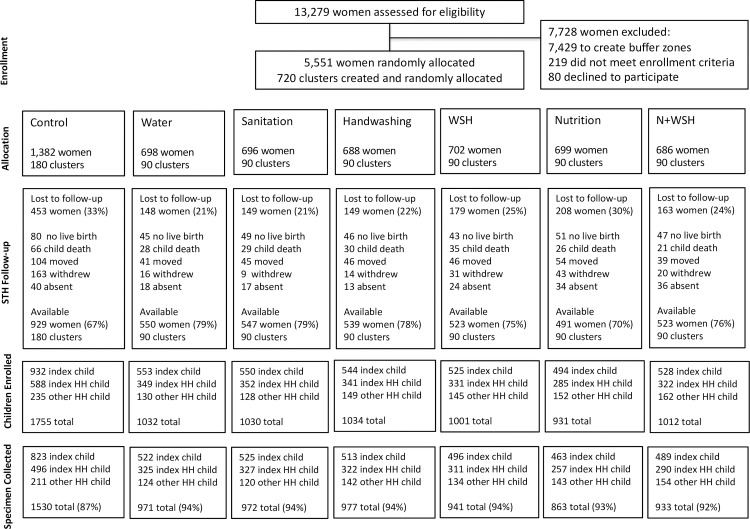
Flowchart of study participation. The STH assessment enrolled (1) the birth cohort born to the enrolled women (referred to as “index child”), (2) children living in the same household as the index child (referred to as “index HH child”) and (3) children living in the same multi-household compound as the index child (referred to as “other HH child”).

Study arms included (1) water treatment: chlorination with sodium dichloroisocyanurate (NaDCC) tablets coupled with safe storage in a narrow-mouth lidded vessel with spigot, (2) sanitation improvements: upgrades to concrete-lined double-pit latrines and provision of child potties and sani-scoops for feces disposal, (3) handwashing promotion: handwashing stations with a water reservoir and a bottle of soapy water mixture at the food preparation and latrine areas, (4) combined water treatment, sanitation and handwashing (WSH), (5) nutrition improvements including exclusive breastfeeding promotion (birth to 6 months), lipid-based nutrient supplements (6–24 months), and age-appropriate maternal, infant, and young child nutrition recommendations (pregnancy to 24 months), (6) nutrition plus combined WSH (N+WSH), and (7) a double-sized control arm with no intervention (Fig 1). Further details of the interventions have been provided elsewhere [22].

**Fig 2:**
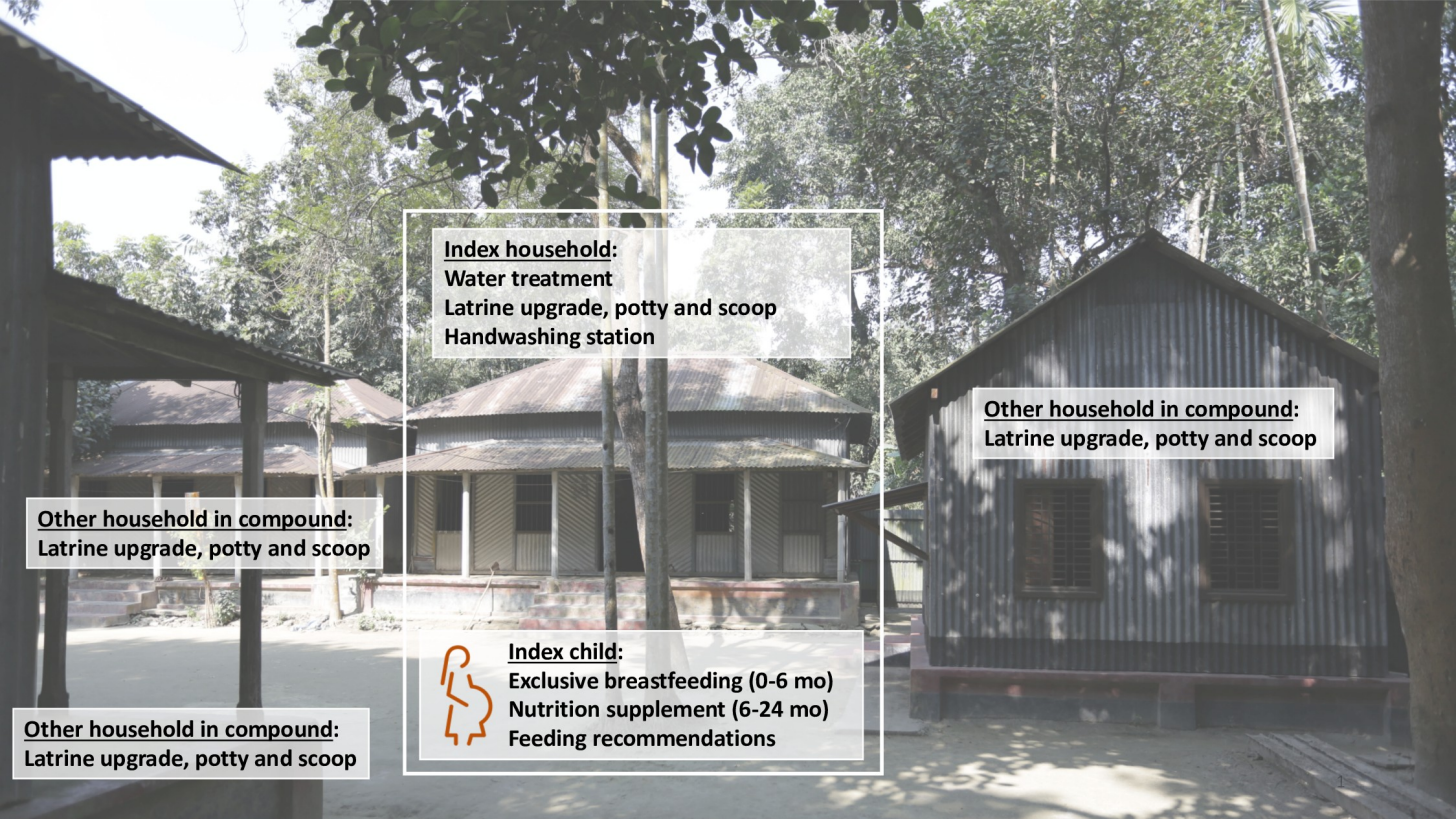
Interventions implemented at index child, index household, and compound levels. Index child refers to the birth cohort born to enrolled pregnant women. Index household refers to the household where the index child lived. Each enrolled compound contained a single index household and an average of 2.5 households total. Index children were the primary intervention recipients of the nutrition intervention delivered in the nutrition and N+WSH arms. Index households were the primary recipients of the water treatment and handwashing interventions; in the water, WSH and N+WSH arms they received water treatment products, and in the handwashing, WSH and N+WSH arms they received handwashing stations. The sanitation intervention was delivered to the entire compound; all households in compounds enrolled in the sanitation, WSH and N+WSH arms received latrine upgrades, child potties and scoops.

The WSH interventions aimed to reduce children’s early-life exposure to fecal pathogens. Bangladeshi households are clustered in compounds shared by extended families; in our study, the compound containing the household where the index child lived (“index household”) had an average of 2.5 households (range: 1–11). The interventions targeted the compound environment as we expected this to be the primary exposure domain for young children [27]. Interventions were delivered at index child, index household and compound levels (Fig 2). The nutrition intervention targeted index children only. The water and handwashing interventions were delivered to the index household. The sanitation intervention provided upgraded latrines, potties and scoops to all households in the compound; as the shared compound courtyard serves as play space for children, we aimed to improve sanitary conditions in this environment with compound-level latrine coverage. Because of the eligibility criterion of having a pregnant woman, enrolled compounds represented approximately 10% of compounds in a given geographical area; as such, we did not provide exclusive community-level latrine coverage.

The delivery of interventions was initiated around the time of index children’s birth. Local women hired and trained as community health promoters visited intervention arm participants on average six times per month to deliver intervention products for free, replenish the supply of consumables (chlorine tablets, soapy water solution, nutrient supplements), resolve hardware problems and encourage adherence to the targeted WSH and nutrition behaviors; health promoters did not visit control arm participants (S4 Text). The health promotion visits and supply of consumable intervention products spanned the full study duration, including the period of the STH assessment. All interventions had high user adherence throughout the study as measured by objective indicators (S4 Text). Further details of intervention adherence have been previously reported [28–30].

### Outcome assessment

We assessed STH infections in children living in WASH Benefits compounds approximately 2.5 years after intervention initiation. Households with no live birth or index child death were excluded from intervention promotion and subsequently follow-up. The following children were eligible to enroll in the STH assessment: (1) all index children (aged 30 months on average at follow-up), (2) up to two other children per enrolled compound aged 3–12 years, living either in the index household or another household in the compound, enrolled in the preferential order of sibling of index child, child living in index household, or child living in another household in the compound, depending on availability.

We did not measure pre-intervention STH prevalence as index children were not born at the time and also because detection of any infections would ethically have required treatment and thus prevented us from testing our hypotheses under the typical conditions of our study population. Randomization was expected to ensure baseline balance in STH outcomes across study arms. Additionally, protozoan infections with *Giardia duodenalis*, *Cryptosporidium spp*., and *Entamoeba histolytica* were measured at baseline among children aged 18–27 months (the anticipated age range for index children at the time of the STH assessment) to assess baseline balance on parasite infections between arms [31].

To measure STH outcomes, field staff distributed sterile containers to primary caregivers of enrolled children, instructed them to collect stool from the following morning’s defecation event, and retrieved the containers on the morning of defecation. If any enrolled child was absent or failed to provide a specimen, field staff returned to the household twice before classifying them as lost to follow-up. After the completion of stool collection in a given compound, all compound members were offered a single dose of albendazole.

Specimens without preservatives were transported on ice to the field laboratory of the International Centre for Diarrhoeal Disease Research, Bangladesh (icddr,b) and analyzed on the same day. Laboratory staff were trained at the icddr,b parasitology laboratory using the Vestergaard Frandsen protocol to perform double-slide Kato-Katz and enumerate ova of *A*. *lumbricoides*, hookworm and *T*. *trichiura*. Two slides were prepared from each stool sample and enumerated within 30 minutes of slide preparation [32]. 10% of slides were counted by two technicians (within the 30 minute-window since slide preparation), and 5% were counted by a senior parasitologist (by sending the slides to the icddr,b parasitology laboratory in Dhaka 0–4 days following the original count at the field laboratory) for quality assurance. Two independent technicians double-entered slide counts into a database.

### Ethics

Primary caregivers of children provided written informed consent. Children aged 7–12 years provided written assent. The protocol was approved by human subjects committees at University of California, Berkeley (2011-09-3652), Stanford University (25863), and the icddr,b (PR-11063). A data safety monitoring committee at icddr,b oversaw procedures.

### Registration

WASH Benefits was registered at ClinicalTrials.gov (NCT01590095) in April 2012 before trial enrolment began in May 2012; this registration lists the trial’s primary and secondary outcomes (diarrhea, child growth). The trial design was published in June 2013 before the STH follow-up began in May 2015 and lists STH under tertiary outcomes in Appendix 3 [22]. The pre-specified analysis plan for the STH outcomes was registered at Open Science Framework (OSF, https://osf.io/v2c8p/) in August 2016 before data analysis began.

### Statistical analysis

#### Outcomes

Our pre-specified outcome measures were the infection prevalence, infection intensity and moderate/heavy infection prevalence for each STH species and for any of the three target species (“any STH”). For each species, we classified stool samples with any ova as positive. We quantified infection intensity in eggs per gram (epg) by multiplying the sum of egg counts from the two duplicated slides by 12 (the equivalent of the standard practice of multiplying slide counts by 24 in single-slide Kato-Katz). We defined moderate/heavy intensity infections based on WHO categories (≥5,000 epg for *A*. *lumbricoides*, ≥2,000 epg for hookworm, and ≥1,000 epg for *T*. *trichiura*) [33]. We assessed the interrater agreement between two independent technicians and between a given technician and the senior parasitologist by calculating the kappa statistic for slides classified as positive [34].

#### Sample size

WASH Benefits was designed to detect effects on child length and diarrhea with a planned sample size of 5040 pregnant women [22]. We assumed that two children per pregnant woman would be eligible for the STH assessment and 70% of children would provide stool. We estimated STH prevalence and intra-class correlation coefficients (ICC) from the literature [35]. With a two-sided α of 0.05, we had 80% power to detect the following relative reductions in prevalence between any intervention arm vs. control: 41% for *A*. *lumbricoides*, 50% for hookworm, 39% for *T*. *trichiura*, and 18% for any STH.

#### Statistical parameters and estimation strategy

We compared STH outcomes in (1) individual and combined water, sanitation, handwashing and nutrition arms vs. controls (primary hypothesis), (2) combined vs. single WSH intervention arms, and (3) N+WSH vs. WSH and nutrition arms. We estimated prevalence ratios (PR), prevalence differences (PD) and fecal egg count reductions (FECR, defined as the epg ratio minus one) between arms. We estimated FECRs using geometric and arithmetic means; while geometric means prevent extreme data points from skewing means, arithmetic means are more sensitive to high infection intensities thought to correlate with higher morbidity burden and transmission [1].

Randomization led to extremely good covariate balance [23], and our primary analysis therefore relied on unadjusted estimates. We estimated the unadjusted parameters using targeted maximum likelihood estimation (TMLE) with influence-curve based standard errors treating clusters as independent units of analysis [36]. Secondary analyses adjusted for pre-specified covariates using data-adaptive machine learning (see analysis plan). Analyses were intention-to-treat as user uptake of interventions was high. As per our pre-specified analysis plan, we did not adjust effect estimates for multiple comparisons as Bonferroni corrections and other multiplicity adjustments can be overcorrections, especially if the outcomes are correlated [37]. All analyses were conducted using R (version 3.3.2).

#### Subgroup analyses

Our primary analysis included all enrolled children of all ages. As different interventions were implemented at index child, index household and compound levels, we also conducted subgroup analyses for the following three categories of children: (1) index children, (2) other children living in index household, and (3) children living in other households in compound. The subgroup analysis for index children was pre-specified and the analysis for other children living in the index household vs. the rest of the compound was added post-hoc. We also conducted a pre-specified subgroup analysis by child age (pre-school-age vs. school-age) as well as additional pre-specified subgroup analyses by deworming status, household size, wealth, housing materials, and baseline sanitation conditions (see analysis plan for details of subgroup analyses).

#### Missing outcomes

Individuals that were lost at follow-up or failed to submit a specimen were classified as missing. To assess if the likelihood of missing data was differential by study arm and/or covariates, we compared the percentage of missing observations between arms and the enrollment characteristics of those with available vs. missing specimens. We also assessed the balance of baseline covariates between arms for households captured at follow-up. We conducted a complete-case analysis and an inverse probability of censoring-weighted (IPCW) analysis re-weighting the measured population to reflect the original enrolled population (see analysis plan) [36].

## Results

### Enrolment

Fieldworkers identified 13279 pregnant women in the study area (Fig 1). Between May 2012-July 2013, we enrolled and randomized 5551 women in 720 clusters; the rest were excluded to create between-cluster buffers (n = 7429), were ineligible (n = 219) or refused (n = 80). At the STH follow-up in May 2015–2016, 1449 women (26%) were lost because of no live birth (n = 361), index child death (n = 235), relocation (n = 375), absence (n = 182) or withdrawal (n = 296) (Fig 1). The control arm had higher attrition (33%) than intervention arms combined (24%) as they had more withdrawals (12% vs. 3%). Among 4102 (74%) available women, we enrolled 7795 children in the STH assessment (an average of 1.8 children per compound and 5.4 compounds per cluster), and we successfully collected stool from 7187 (92%) (Fig 1). Stool recovery was somewhat lower in controls (87%) than in intervention arms (94%). Household-level enrolment covariates measured at baseline were balanced between arms for index households captured at follow-up (Table 1) and for those with vs. without specimens (S1 Table). The prevalence of protozoan parasites measured among children aged 18–27 months at baseline was balanced between arms [31].

**Table 1:** Household-level enrolment characteristics by intervention group measured at baseline (for index households that participated in STH assessment at follow-up). ^a^

	Control	Water	Sanitation	Handwashing	WSH	Nutrition	N+WSH
No. of women:	(N = 929)	(N = 550)	(N = 547)	(N = 539)	(N = 523)	(N = 491)	(N = 523)
**Maternal**							
Age, mean (range)	24 (15–43)	24 (15–43)	24 (15–41)	24 (15–60)	25 (15–44)	24 (15–45)	24 (14–43)
Years of education, mean (range)	6 (0–15)	6 (0–14)	6 (0–17)	6 (0–16)	6 (0–14)	6 (0–16)	6 (0–14)
**Paternal**							
Years of education, mean (range)	5 (0–16)	5 (0–16)	5 (0–17)	5 (0–16)	5 (0–16)	5 (0–16)	5 (0–16)
Works in agriculture, % (n)	31.4 (292)	31.5 (173)	30.7 (168)	37.5 (202)	31.0 (162)	33.6 (165)	31.2 (163)
**Household**							
Number of persons, mean (range)	5 (2–17)	5 (2–23)	5 (2–17)	5 (2–22)	5 (1–14)	5 (2–18)	5 (2–14)
Has electricity, % (n)	57.9 (538)	62.7 (345)	60.5 (331)	59.7 (322)	63.1 (330)	61.3 (301)	60.6 (317)
Has a cement floor, % (n)	10.0 (93)	12.0 (66)	12.1 (66)	8.0 (43)	10.7 (56)	8.6 (42)	12.1 (63)
Acres of agricultural land owned, mean (range)	0.1 (0.0–2.5)	0.1 (0.0–2.4)	0.1 (0.0–3.2)	0.1 (0.0–2.6)	0.2 (0.0–3.1)	0.2 (0.0–2.8)	0.2 (0.0–8.9)
**Drinking water**							
Shallow tubewell primary water source, % (n)	76.5 (711)	73.5 (404)	75.1 (411)	70.3 (379)	79.0 (413)	75.2 (369)	74.0 (387)
Stored water observed at home, % (n)	46.6 (433)	51.1 (281)	47.4 (259)	48.8 (263)	41.5 (217)	41.8 (205)	48.0 (251)
Reported treating water yesterday, % (n)	0.3 (3)	0.2 (1)	0.0 (0)	0.2 (1)	0.0 (0)	0.0 (0)	0.4 (2)
**Sanitation**							
Daily defecating in the open, % (n)							
Adult men	7.2 (67)	5.3 (29)	6.6 (36)	9.8 (53)	6.5 (34)	7.3 (36)	7.5 (39)
Adult women	4.7 (44)	2.6 (14)	4.2 (23)	5.2 (28)	4.0 (21)	5.30 (26)	3.8 (20)
Children: 8-<15 years (N = 1743)	10.2 (38)	9.5 (21)	8.9 (22)	15.5 (37)	8.3 (19)	8.3 (17)	9.5 (22)
Children: 3-<8 years (N = 2179)	40.0 (197)	36.0 (111)	37.2 (109)	37.7 (110)	35.5 (99)	35.1 (85)	36.3 (99)
Children: 0-<3 years (N = 848)	80.5 (157)	85.6 (89)	81.1 (86)	85.5 (100)	78.0 (92)	82.5 (85)	88.6 (93)
Latrine, % (n)							
Owned ^b^	53.4 (496)	52.9 (291)	53.4 (292)	54.6 (294)	53.0 (277)	54.2 (266)	54.1 (283)
Concete slab	90.4 (840)	92.7 (510)	88.3 (483)	89.6 (483)	90.1 (471)	90.0 (440)	90.3 (472)
Functional water seal	25.3 (235)	26.4 (145)	26.0 (142)	25.4 (137)	21.0 (110)	26.5 (130)	22.6 (118)
Visible stool on slab or floor	48.6 (451)	44.9 (247)	44.8 (245)	43.8 (236)	52.6 (275)	46.2 (227)	49.3 (258)
Owned a potty, % (n)	3.4 (32)	3.8 (21)	3.8 (21)	5.0 (27)	3.6 (19)	5.3 (26)	4.8 (25)
Human feces observed in, % (n)							
House	9.0 (84)	9.6 (53)	7.7 (42)	10.6 (57)	7.1 (37)	7.1 (35)	6.9 (36)
Child's play area	1.4 (13)	1.1 (6)	0.9 (5)	1.1 (6)	0.8 (4)	0.6 (3)	1.2 (6)
**Handwashing**							
Has within 6 steps of latrine, % (n)							
Water	12.8 (119)	12.0 (66)	11.9 (65)	8.5 (46)	8.2 (43)	8.6 (42)	11.7 (61)
Soap	5.5 (51)	6.9 (38)	7.5 (41)	4.8 (26)	4.6 (24)	4.5 (22)	5.9 (31)
Has within 6 steps of kitchen, % (n)							
Water	8.5 (79)	6.6 (36)	7.3 (40)	5.8 (31)	8.2 (43)	9.1 (45)	8.8 (46)
Soap	2.4 (22)	2.2 (12)	2.0 (11)	2.0 (11)	2.1 (11)	3.9 (19)	3.3 (17)

^a^ Household-level characteristics are only available for index households as we did not collect information on other households in the compound.

^b^ Households in these communities who do not own a latrine typically share a latrine with extended family members who live in the same compound.

### Child-level characteristics

Average age at follow-up was 30 months (range: 22–38) for index children and 7 years (range: 3–12) for non-index children. Caregivers reported that 60% of index children and 67% of non-index children had been dewormed in the six months prior to our data collection. For index children, the predominant source (86%) of deworming drugs was individual purchase at a pharmacy. For non-index children, the predominant sources of deworming drugs were schools (48%) and pharmacies (44%), and approximately half of children were reported to be dewormed through the MDA program. The average time since deworming was 9 weeks (range: 0–24) for both index and non-index children. The percentage of dewormed children and the average time since deworming was balanced across trial arms (Table 2). Caregivers reported that 26% of index children and 8% of non-index children ingested soil in the last week, and 15% of index children and 25% of non-index children were observed to be wearing shoes.

**Table 2:** Child-level characteristics by intervention group measured at follow-up.

	Control	Water	Sanitation	Handwashing	WSH	Nutrition	N+WSH
Index children	N = 823	N = 522	N = 525	N = 513	N = 496	N = 463	N = 489
Male, % (n)	49.1 (404)	49.8 (260)	49.7 (261)	48.0 (246)	51.8 (257)	51.2 (237)	46.6 (228)
Age in months, mean (range)	30 (25–37)	30 (25–38)	30 (25–35)	30 (24–37)	30 (22–35)	30 (26–38)	30 (25–35)
Deworming within last 6 months, % (n)	64.0 (527)	61.9 (323)	58.5 (307)	59.5 (305)	57.5 (285)	58.5 (271)	59.5 (291)
Deworming as part of MDA, % (n) ^a^	7.4 (39)	9.3 (30)	7.2 (22)	9.8 (30)	5.3 (15)	6.6 (18)	4.8 (14)
Deworming source, % (n) ^a^							
Pharmacy	87.7 (462)	83.0 (268)	89.3 (274)	79.3 (242)	87.0 (248)	87.8 (238)	88.0 (256)
Clinic	10.8 (57)	14.6 (47)	8.8 (27)	19.0 (58)	12.6 (36)	11.1 (30)	10.7 (31)
School	1.5 (8)	1.9 (6)	1.0 (3)	1.6 (5)	0.4 (1)	0.7 (2)	1.0 (3)
Weeks since deworming, mean (range) ^a^	9 (1–24)	9 (1–24)	9 (1–22)	9 (1–21)	9 (1–23)	10 (0–24)	9 (1–24)
Child ingested soil within last week, % (n)	36.7 (302)	19.2 (100)	29.5 (155)	19.5 (100)	25.6 (127)	21.8 (101)	19.0 (93)
Child wearing shoes, % (n)	16.5 (136)	15.7 (82)	14.3 (75)	16.2 (83)	14.5 (72)	13.2 (61)	16.8 (82)
Non-index children	N = 707	N = 449	N = 447	N = 464	N = 445	N = 400	N = 444
Male, % (n)	45.3 (320)	47.9 (215)	53.5 (239)	47.4 (220)	50.3 (224)	48.0 (192)	50.0 (222)
Age in years, mean (range)	7 (4–12)	7 (4–12)	7 (4–12)	7 (3–12)	7 (3–12)	7 (4–12)	7 (4–12)
School-age, % (n)	89.0 (629)	87.5 (393)	89.7 (401)	88.6 (411)	87.9 (391)	85.3 (341)	88.1 (391)
Deworming within last 6 months, % (n)	70.2 (496)	67.7 (304)	74.1 (331)	66.4 (308)	63.8 (284)	63.3 (253)	62.8 (279)
Deworming as part of MDA, % (n) ^a^	48.2 (239)	54.0 (164)	50.2 (166)	56.2 (173)	51.1 (145)	48.2 (122)	45.5 (127)
Deworming source, % (n) ^a^							
Pharmacy	47.6 (236)	40.5 (123)	42.9 (142)	39.3 (121)	41.6 (118)	45.5 (115)	47.7 (133)
Clinic	7.3 (36)	9.2 (28)	5.7 (19)	9.7 (30)	9.5 (27)	11.1 (28)	6.8 (19)
School	45.0 (223)	50.0 (152)	50.1 (167)	51.0 (157)	48.9 (139)	42.7 (108)	45.5 (127)
Weeks since deworming, mean (range) ^a^	9 (0–24)	9 (1–24)	9 (1–24)	9 (1–24)	9 (1–22)	9 (0–20)	9 (1–24)
Child ingested soil within last week, % (n)	16.0 (113)	2.7 (12)	11.0 (49)	2.4 (11)	9.7 (43)	5.3 (21)	6.3 (28)
Child wearing shoes, % (n)	28.2 (199)	26.3 (118)	26.0 (116)	23.7 (110)	20.7 (92)	26.0 (104)	24.6 (109)

^a^ Among children reported to be dewormed within last 6 months.

### Infection prevalence and intensity

STH prevalence among all children in the control arm was 36.8% (n = 563) for *A*. *lumbricoides*, 9.2% (n = 142) for hookworm, 7.5% (n = 115) for *T*. *trichiura*, and 43.4% (n = 664) for any STH. The geometric mean egg count among controls was 5.2 epg (143.4 epg among positive samples) for *A*. *lumbricoides*, 0.6 epg (119.8 epg among positive samples) for hookworm and 0.4 epg (115.4 epg among positive samples) for *T*. *trichiura*. Most infections were low-intensity. Moderate/heavy infection prevalence among controls was 4.2% (n = 65) for *A*. *lumbricoides*, 0.1% (n = 2) for hookworm and 0.4% (n = 6) for *T*. *trichiura*. The ICC for any STH infection was 0.18 for children within the same compound (0.18 for *A*. *lumbricoides*, 0.03 for hookworm and 0.21 for *T*. *trichiura*) and 0.08 for children within the same cluster of compounds (0.08 for *A*. *lumbricoides*, 0.06 for hookworm and 0.13 for *T*. *trichiura*). The quality control measures indicated high interrater reliability (S5 Text).

### Interventions vs. control

**Fig 3:**
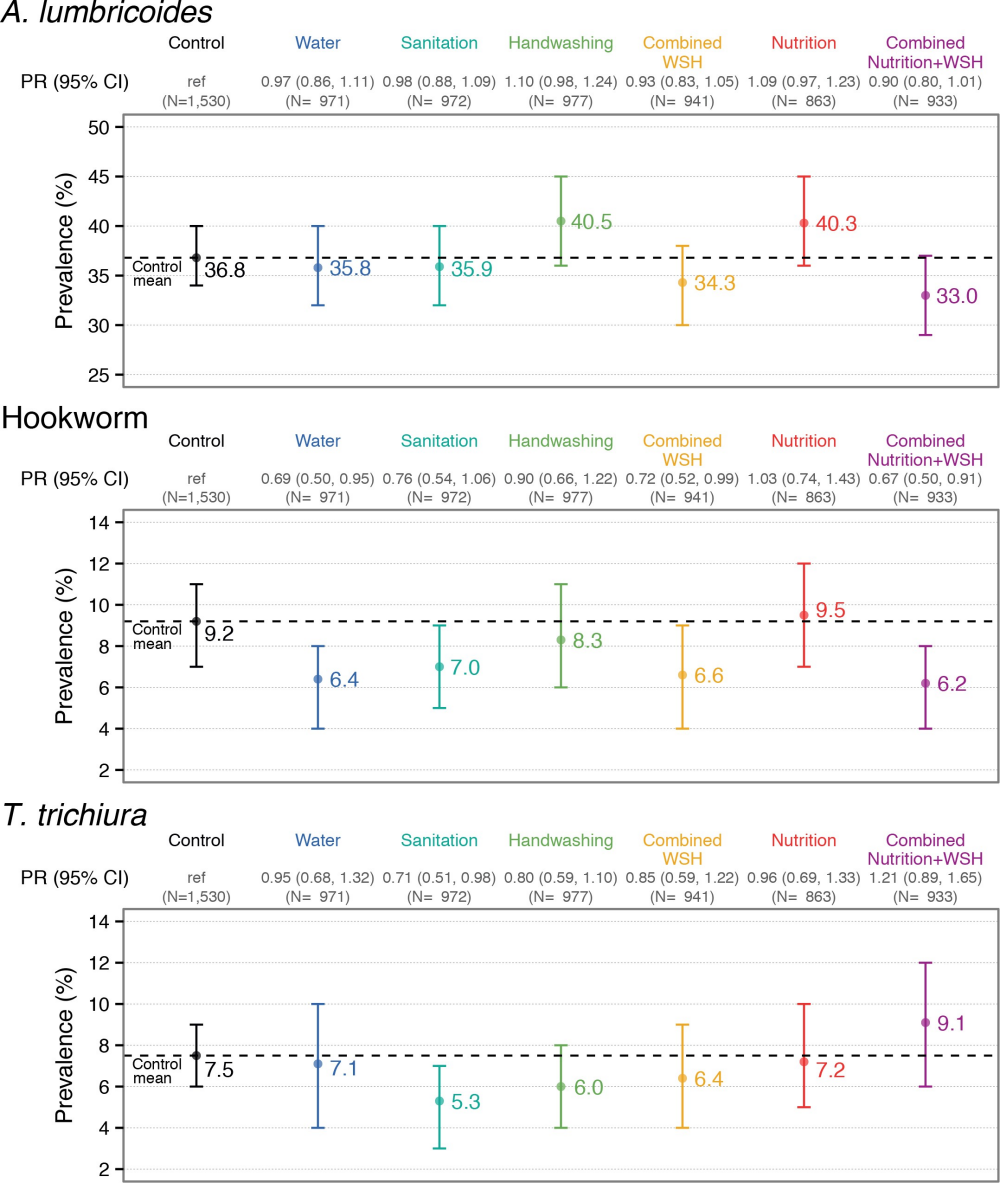
Prevalence and prevalence ratio (PR) for *A*. *lumbricoides*, hookworm, and *T*. *trichiura* infection in children aged 2–12 years measured with double-slide Kato-Katz 2.5 years after intervention initiation. Vertical bars indicate 95% confidence intervals.

Among all enrolled children, the single water intervention reduced hookworm prevalence by 31% (PR = 0.69 (0.50, 0.95), PD = -2.83 (-5.16, -0.50)) from a control prevalence of 9.2% but had no effect on other STH (Fig 3, Table 3, S2 Table). The sanitation intervention reduced *T*. *trichiura* prevalence by 29% (PR = 0.71 (0.52, 0.98), PD = -2.17 (-4.10, -0.24)) from a control prevalence of 7.5% and achieved a similar borderline reduction on hookworm but had no effect on *A*. *lumbricoides*. Single handwashing or nutrition interventions did not reduce the prevalence of any STH compared to controls; there was a borderline increase in *A*. *lumbricoides* prevalence in these arms (Fig 3, Table 3, S2 Table).

**Table 3:** STH prevalence ratio for intervention vs. control arms for index children, other children in index household and children in other households in compound, unadjusted analysis.

	All observations			Index children ^a^			Other children in index household ^b^			Children in non-index households ^c^		
	N	Prev.	Prevalence ratio	N	Prev.	Prevalence ratio	N	Prev.	Prevalence ratio	N	Prev.	Prevalence ratio
***A*. *lumbricoides***												
Control	1530	36.8%		823	31.3%		496	44.4%		211	40.3%	
Water	971	35.8%	0.97 (0.86, 1.11)	522	28.0%	0.89 (0.74, 1.07)	325	43.4%	0.98 (0.82, 1.18)	124	49.2%	1.22 (0.91, 1.64)
Sanitation	972	35.9%	0.98 (0.88, 1.09)	525	30.3%	0.97 (0.83, 1.13)	327	42.8%	0.98 (0.84, 1.13)	120	41.7%	0.96 (0.73, 1.27)
Handwashing	977	40.5%	1.10 (0.98, 1.24)	513	36.5%	1.16 (0.97, 1.40)	322	45.7%	1.05 (0.89, 1.23)	142	43.7%	1.05 (0.80, 1.39)
WSH	941	34.3%	0.93 (0.83, 1.05)	496	28.8%	0.92 (0.78, 1.09)	311	40.5%	0.93 (0.78, 1.12)	134	40.3%	1.00 (0.74, 1.34)
Nutrition	863	40.3%	1.10 (0.98, 1.23)	463	35.0%	1.12 (0.96, 1.29)	257	49.0%	1.14 (0.95, 1.37)	143	42.0%	1.14 (0.84, 1.54)
Nutrition + WSH	933	33.0%	0.90 (0.80, 1.01)	489	26.4%	0.84 (0.72, 0.99)	290	41.0%	0.95 (0.80, 1.13)	154	39.0%	1.02 (0.75, 1.40)
**Hookworm**												
Control	1530	9.2%		823	3.6%		496	16.1%		211	14.7%	
Water	971	6.4%	0.69 (0.50, 0.95)	522	3.4%	0.95 (0.52, 1.71)	325	9.5%	0.59 (0.39, 0.90)	124	10.5%	0.75 (0.37, 1.51)
Sanitation	972	7.0%	0.76 (0.54, 1.06)	525	2.7%	0.73 (0.35, 1.51)	327	12.5%	0.78 (0.52, 1.17)	120	10.8%	0.78 (0.38, 1.58)
Handwashing	977	8.3%	0.90 (0.66, 1.22)	513	3.5%	0.96 (0.49, 1.90)	322	13.4%	0.84 (0.61, 1.16)	142	14.1%	0.95 (0.54, 1.69)
WSH	941	6.6%	0.71 (0.52, 0.99)	496	3.0%	0.83 (0.49, 1.40)	311	11.3%	0.70 (0.46, 1.08)	134	9.0%	0.63 (0.36, 1.09)
Nutrition	863	9.5%	1.03 (0.74, 1.43)	463	5.0%	1.36 (0.72, 2.59)	257	14.0%	0.90 (0.62, 1.30)	143	16.1%	1.37 (0.78, 2.42)
Nutrition + WSH	933	6.2%	0.67 (0.50, 0.91)	489	3.1%	0.84 (0.51, 1.38)	290	9.7%	0.62 (0.41, 0.94)	154	9.7%	0.58 (0.28, 1.20)
***T*. *trichiura***												
Control	1530	7.5%		823	5.2%		496	9.5%		211	11.8%	
Water	971	7.1%	0.95 (0.68, 1.32)	522	4.6%	0.88 (0.54, 1.44)	325	9.5%	1.02 (0.63, 1.66)	124	11.3%	0.86 (0.42, 1.78)
Sanitation	972	5.3%	0.71 (0.52, 0.98)	525	3.2%	0.62 (0.38, 1.00)	327	7.3%	0.81 (0.49, 1.35)	120	9.2%	0.72 (0.41, 1.27)
Handwashing	977	6.0%	0.80 (0.59, 1.10)	513	3.7%	0.71 (0.41, 1.21)	322	9.3%	0.98 (0.61, 1.59)	142	7.0%	0.72 (0.34, 1.51)
WSH	941	6.4%	0.85 (0.59, 1.22)	496	5.0%	0.96 (0.63, 1.47)	311	8.0%	0.87 (0.54, 1.41)	134	7.5%	0.69 (0.32, 1.49)
Nutrition	863	7.2%	0.96 (0.69, 1.33)	463	4.5%	0.87 (0.55, 1.37)	257	8.6%	0.92 (0.57, 1.50)	143	13.3%	1.31 (0.61, 2.81)
Nutrition + WSH	933	9.1%	1.21 (0.89, 1.65)	489	6.3%	1.21 (0.79, 1.86)	290	12.4%	1.33 (0.88, 2.02)	154	11.7%	1.04 (0.52, 2.08)
**Any STH**												
Control	1530	43.4%		823	35.2%		496	54.2%		211	49.8%	
Water	971	42.5%	0.98 (0.87, 1.10)	522	33.0%	0.94 (0.79, 1.11)	325	52.6%	0.98 (0.85, 1.14)	124	56.5%	1.14 (0.89, 1.46)
Sanitation	972	40.6%	0.94 (0.84, 1.04)	525	32.6%	0.92 (0.79, 1.07)	327	50.2%	0.95 (0.82, 1.09)	120	50.0%	0.97 (0.75, 1.24)
Handwashing	977	46.3%	1.07 (0.96, 1.19)	513	38.8%	1.10 (0.93, 1.31)	322	54.7%	1.02 (0.89, 1.17)	142	54.2%	1.07 (0.85, 1.35)
WSH	941	39.3%	0.91 (0.81, 1.01)	496	32.3%	0.92 (0.79, 1.06)	311	47.6%	0.90 (0.76, 1.06)	134	46.3%	0.93 (0.70, 1.23)
Nutrition	863	45.1%	1.04 (0.93, 1.16)	463	38.2%	1.08 (0.95, 1.24)	257	53.7%	1.02 (0.86, 1.21)	143	51.7%	1.14 (0.88, 1.48)
Nutrition + WSH	933	38.8%	0.89 (0.81, 0.99)	489	30.1%	0.85 (0.74, 0.98)	290	49.3%	0.93 (0.80, 1.08)	154	46.8%	0.95 (0.74, 1.23)

^a^ Children born to enrolled pregnant women following enrolment. The nutrition intervention was delivered to index children only.

^b^ Other children living in index child’s household (index household). These include siblings of index children and children from other mothers in the same household. The water and handwashing interventions were delivered to index households only.

^c^ Children living in other households in index child’s compound. The sanitation intervention was delivered to all households in the compound.

Combined WSH reduced hookworm prevalence by 29% (PR = 0.71 (0.52, 0.99), PD = -2.63 (-4.95, -0.31)) and N+WSH by 33% (PR = 0.67 (0.50, 0.91), PD = -3.00 (-5.14, -0.85)) compared to controls. WSH and N+WSH also marginally reduced *A*. *lumbricoides* by 7–10% compared to controls (WSH: PR = 0.93, (0.83, 1.05), PD = -2.47 (-6.54, 1.60); N+WSH: PR = 0.90 (0.81, 1.01), PD = -3.79 (-7.74, 0.17)) but had no effect on *T*. *trichiura* (Fig 3, Table 3, S2 Table).

### Combined vs. single interventions

Compared with single water, sanitation and handwashing interventions, combined WSH reduced *A*. *lumbricoides* more than handwashing alone (PR = 0.85, (0.75, 0.96), PD = -6.21 (-10.96, -1.46); this reflects the increased *A*. *lumbricoides* prevalence in the handwashing arm. We found no other benefit from combined WSH vs. its individual components (Fig 3, S3 Table). Combined N+WSH reduced *A*. *lumbricoides* and hookworm prevalence compared to nutrition alone (*A*. *lumbricoides*: PR = 0.82 (0.72, 0.93), PD = -7.31 (-12.06, -2.57)); hookworm: PR = 0.65 (0.47, 0.91), PD = -3.29 (-5.96, -0.61)) but did not achieve any reduction compared to WSH (Fig 3, S4 Table).

### Other effects

**Fig 4:**
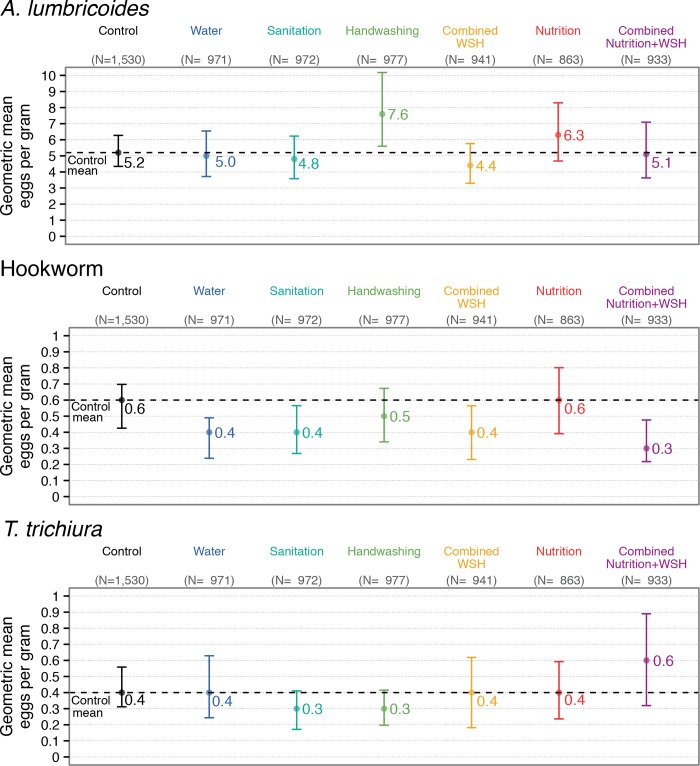
Geometric mean eggs per gram for *A*. *lumbricoides*, hookworm and *T*. *trichiura* in children aged 2–12 years measured with double-slide Kato-Katz 2.5 years after intervention initiation.

Effects on any STH closely reflected *A*. *lumbricoides* results due to the high prevalence of *A*. *lumbricoides* compared to the other two species (Fig 3, S2–S4 Tables). Interventions did not affect the prevalence of moderate/heavy infections (S5–S7 Tables) but we had low power for these rare outcomes. Effects on infection intensity were similar to effects on prevalence, except for a modest reduction in *T*. *trichiura* intensity from handwashing (Fig 4, S8–S10 Tables). Arithmetic means yielded similar results with wider confidence intervals (S8–S10 Tables). Unadjusted, adjusted and IPCW estimates were similar (S2–S10 Tables).

### Subgroup analyses

Subgroup analyses on index children, other children in the index household and children in other households in the compound yielded findings consistent with those using pooled data from all children. Non-index children had higher infection prevalence (Table 3), consistent with previous evidence on these age groups [1]. The water intervention, which was implemented in the index household and showed a reduction in hookworm when using data from all enrolled children, substantially reduced hookworm among the older children living in the index household (PR = 0.59 (0.39, 0.90)) but not among index children themselves who had lower infection prevalence and also might have consumed less water due to their younger age (PR = 0.95 (0.52, 1.71)), nor among children in other households in the compound whose own households did not receive the water intervention (PR = 0.75 (0.37, 1.51)). The handwashing intervention, which was implemented in the index household and did not achieve a reduction when using data from all children, also did not achieve a reduction among children living in the index household (Table 3). Similarly, the nutrition intervention, which was provided to index children only and did not achieve a reduction when using data from all enrolled children, also did not achieve a reduction among index children (Table 3). As expected, the reduction on hookworm and *T*. *trichiura* from the compound-level sanitation intervention was similar among all children in the compound (Table 3); however, most confidence intervals crossed the null, reflecting the small sample sizes of the subgroups. Point estimates suggested that the WSH and N+WSH interventions, which reduced hookworm among all children, achieved similar reductions in all three subgroups of children but, once again, most confidence intervals crossed the null due to small sample size (Table 3). Our full set of subgroup findings are reported elsewhere (https://osf.io/v2c8p/); we note that these analyses should be considered exploratory as they had limited statistical power.

## Discussion

### Effect of water treatment

In all intervention arms that included water treatment (the individual water treatment, WSH and N+WSH arms), we found a significant reduction in hookworm but not in *A*. *lumbricoides* or *T*. *trichiura*. These findings suggest possible waterborne hookworm transmission, and chlorine water treatment with safe storage is effective in reducing this transmission. While infections of *A*. *lumbricoides* or *T*. *trichiura* are transmitted by ingesting embryonated ova, hookworm larvae infect hosts by penetrating skin; however *A*. *duodenale* can also be transmitted by ingesting larvae [1]. A study using molecular diagnostics on infant stool samples in Dhaka detected *A*. *duodenale* several times more frequently than *N*. *americanus*, suggesting that *A*. *duodenale* could be the dominant hookworm species in the region [38]. STH ova have been detected in drinking water in low-income countries [39], suggesting a potential reservoir. A systematic review reported that boiling and filtering water was associated with reduced STH infection [8]. While chlorination is generally considered ineffective against STH ova [40], fragile hookworm ova and larvae could be more chlorine-susceptible than the hardier ova of *A*. *lumbricoides* or *T*. *trichiura*. The safe storage container with a narrow mouth and lid would also reduce STH contamination of stored drinking water by eliminating contact with hands, which are known reservoirs of ova and larvae [41]. An observational study found increased STH infection associated with unhygienic water storage [42]. Storage could also allow eggs to settle out of the water column before consumption [40]. However, while safe storage should similarly affect all three STH species, *A*. *lumbricoides* or *T*. *trichiura* were not reduced by our water intervention, potentially suggesting that the hookworm reduction is due to chlorine rather than safe storage; there are scarce data on the effectiveness of chlorine on hookworm. Alternatively, waterborne transmission could be more important for hookworm in this setting than for *A*. *lumbricoides* or *T*. *trichiura*.

### Effect of sanitation

The WASH Benefits sanitation intervention with concrete-lined double-pit latrines, potties and scoops for feces management reduced *T*. *trichiura* and achieved a borderline reduction in hookworm but had no effect on *A*. *lumbricoides*. While the other two arms with a sanitation component (WSH, N+WSH) reduced hookworm to a similar degree as the single sanitation intervention, *T*. *trichiura* was not affected in these arms; the reduction in the sanitation arm for this species could thus be a chance finding and should be interpreted cautiously. Two previous sanitation trials in India found no impact on STH; however, both studies entailed community-level programs with relatively low adherence [12,13]. WASH Benefits implemented a compound-level intervention with high adherence. At follow-up, >95% of respondents in the sanitation, WSH and N+WSH arms had a latrine with a functional water seal compared to <25% of controls [30]. In structured observations, >90% of adults in sanitation arms used a hygienic latrine vs. 40% of controls [30]. Low adherence is therefore unlikely to explain any lack of intervention impact. However, it is possible that structured observations overestimated actual latrine use due to respondent reactivity [43]. Also, children continued open defecation despite sanitation access; only 37–54% of young children in sanitation arms were observed to defecate in a latrine or potty vs. 32% of controls [30]. Finally, WASH Benefits intervened on roughly 10% of compounds within a geographical area and did not implement exclusive community-level latrine coverage. Bangladesh has a high population density, and STH ova from surrounding non-study compounds could have entered intervention compounds on shoes/soles of compound residents or via surface runoff. Bangladeshi families also use soil from outside the compound to coat walls and courtyards. Community-level sanitation coverage may be more instrumental in improving child health than individual household sanitation in rural settings [44,45]; it is possible that high community-level sanitation coverage is needed to more broadly impact STH infections.

The lack of sanitation impact on *A*. *lumbricoides* could also be due to its prolonged survival in soil, providing a persistent reservoir to sustain infection [15]. Hookworm larvae survive in soil for weeks and *T*. *trichiura* ova for months [46]. In contrast, *A*. *lumbricoides* ova can survive in soil for several years in warm and saturated conditions [15]. A pilot assessment among study households found ova of *A*. *lumbricoides* in 67% and *T*. *trichiura* in 36% of courtyard soil samples; hookworm ova were not detected in soil (the protocol was not designed to detect hookworm larvae). Among positive samples, 70% of *A*. *lumbricoides* and 88% of *T*. *trichiura* ova developed larvae when incubated (i.e., were viable) [47]. Soil from study households also had human and animal fecal markers and high concentrations of fecal indicator bacteria, suggesting heavy fecal contamination [24,48]. Any reductions in fecal input into the environment through sanitation interventions would plausibly be reflected in a more immediate reduction in infections with hookworm and *T*. *trichiura* whose larvae/ova are shorter-lived in the environment than those of *A*. *lumbricoides*, which is consistent with our findings. The effect of sanitation on *A*. *lumbricoides* infections may not be apparent until existing ova in the environment from pre-intervention contamination are naturally inactivated.

### Effect of handwashing

Handwashing did not reduce STH infection except for a modest reduction in *T*. *trichiura* intensity. Previous hygiene programs have reduced STH infections [9–11]. Two of the previous studies were conducted in schools [9,10], which may have fewer sources of fecal contamination than the domestic environment and therefore lower risk of re-contamination of hands following handwashing. It is also possible that these studies achieved better handwashing than WASH Benefits, potentially because our promotion primarily targeted caregivers rather than children, some of whom were too young to wash their own hands. A study in Bangladesh found *A*. *lumbricoides* ova in 51%, *T*. *trichiura* ova in 23% and hookworm larvae in 26% of fingernails [41], and nail clipping reduced parasite infections among Ethiopian children [11]. While our intervention promotion mentioned washing with soap under fingernails, the practices adopted by participants may not have been sufficient to remove ova/larvae from under nails. Indeed, hand observations showed that children in the handwashing arm had cleaner finger pads and palms than children in the control arm but there was little difference in the visual cleanliness of fingernails [49]. This could also explain why the *T*. *trichiura* intensity but not prevalence was reduced in the handwashing arm; if the intervention reduced but did not eliminate *T*. *trichiura* ova on hands, this could lead to a reduced worm burden without affecting prevalence.

### Effect of combined WSH interventions

We found no added benefit from combining WSH interventions compared to individual interventions. While we did not power the study to statistically detect differences between combined vs. individual intervention arms, the effect estimates suggest that combined WSH and N+WSH achieved a similar magnitude of reduction in hookworm prevalence as the individual water and sanitation interventions. One possible explanation is that combined interventions might have lower user adherence as they require more complex behavior change [50]. However, adherence indicators were similar between individual and combined intervention arms in our study [30]. It is also possible that the primary barrier of sanitation (reducing spread of ova into water sources) and the secondary barrier of water treatment (reducing ingestion of ova/larvae) were operating on the same waterborne transmission pathway and there was thus no benefit from combining them. Nonetheless, combined WSH was the only intervention that achieved a small (albeit borderline non-significant) reduction in *A*. *lumbricoides* compared to controls.

### Effect of nutrition

Lipid-based nutrient supplements, breastfeeding and complementary feeding promotion did not reduce STH prevalence/intensity alone or in combination with WSH, even when the analysis was restricted to index children directly receiving this intervention. There was a borderline increase in *A*. *lumbricoides* prevalence in the nutrition arm. A recent study found increased hookworm in school children receiving micronutrient-fortified rice [51]. Other studies found STH reductions from nutritional supplements [20]. WASH Benefits showed improved child growth and micronutrient status and reduced anemia in the nutrition and N+WSH arms [23,52]. One reason for the lack of STH reduction despite improved nutrition could be the dual direction of possible biological associations between nutrition and STH infection. Breastfeeding and improved nutrition could decrease infection risk by improving immune response and cell repair; conversely, it could increase risk by making nutrients available to helminths [20,21]. Chronic heavy STH infections can lead to malnutrition and growth faltering [46]. Hookworm reductions in the water and WSH arms were not reflected by improved growth in these arms. However, children in the WSH arm had a borderline reduction in anemia and iron deficiency [52], which would be consistent with the reduction in hookworm prevalence in this arm. Nutritional outcomes are likely interrelated with myriad causal effects and the impact of STH on growth should be further assessed.

### Findings in the context of MDA

STH control policies, which currently emphasize MDA programs, could be strengthened by complementing these with water, sanitation and hygiene interventions [53]. Approximately two thirds of children in our study had received deworming within the 6 months prior to our STH assessment, and among these, the average time since deworming was approximately two months. It is possible that, had more time elapsed since the last reported deworming episode at the time of our assessment, allowing time for more prevalent reinfection, our interventions might have demonstrated a larger difference between intervention and control groups. Indeed, the majority of infections we detected were low-intensity, suggesting that deworming successfully reduced the prevalence of heavy infections that drive the morbidity burden [1]. However, despite recent deworming, 43% of children in the control arm were infected with STH (mostly *A*. *lumbricoides*), demonstrating ongoing transmission and suggesting that MDA alone is unlikely to break STH transmission in this setting.

Against this backdrop, we found a 30% relative reduction in hookworm prevalence from water treatment and combined WSH interventions, as well as a borderline reduction of similar size from sanitation improvements. While the reductions were comparable in magnitude to the reductions in child diarrhea and protozoan infections achieved by WASH Benefits [23,31] and other water treatment and hygiene trials in low-income countries [54,55], they are small compared to the typical cure rates from deworming [3,26]. However, while re-infection rates following deworming can be as high as 94% within 12 months of drug administration [5], the effects we report were observed 2.5 years after intervention initiation, suggesting sustained reductions in environmental transmission in a population receiving biannual MDA.

It is also possible that the effect of water, sanitation and hygiene on STH depends on background transmission intensity. In our study, WSH interventions had more pronounced effect on hookworm, which was relatively rare (9% control prevalence), than on *A*. *lumbricoides*, which was more common (37% control prevalence). This is consistent with a school-based trial in Kenya that found reduction in *A*. *lumbricoides* (9–14% prevalence in the study population) but not the more prevalent hookworm (28–29% prevalence) from a combined water, sanitation and hygiene intervention [18]. These findings suggest that in settings where deworming has been successfully implemented to reduce infection intensity and morbidity, water, sanitation and hygiene interventions can complement MDA programs in striving toward elimination by interrupting environmental transmission.

### Limitations

We measured STH infection using Kato-Katz, which has poor sensitivity when infection intensity is low. A systematic review demonstrated a sensitivity of 55% for *A*. *lumbricoides*, 53% for hookworm, 80% for *T*. *trichiura* for double-slide Kato-Katz for low-intensity infections [56]. As 95% of infections in our study were low-intensity, this could yield substantial false negatives in our outcome measurements. Recently developed sensitive nucleic acid-based diagnostics can detect infections that are missed by Kato-Katz [57,58]. We preserved an additional stool aliquot for validation analysis by quantitative polymerase chain reaction (qPCR). Preliminary analyses in a validation study using a subset of our specimens suggest that double-slide Kato-Katz had low to moderate sensitivity for all three STH while it had moderate specificity for *A*. *lumbricoides* and high specificity for *T*. *trichiura* and hookworm (Benjamin-Chung et al. 2019, *in prep*). Assuming non-differential misclassification by arm, imperfect sensitivity and specificity would bias our estimated intervention effects toward the null (S6 Text). If the interventions reduced infection intensity, imperfect sensitivity could also lead to differential misclassification by arm, where a larger proportion of cases in the intervention arms would go undetected by Kato-Katz and intervention effects would therefore be biased away from the null.

Also, WASH Benefits was designed around its primary outcomes (length-for-age Z-score and diarrhea) so there was only sufficient statistical power to detect relatively large effects on hookworm and *T*. *trichiura* given their low prevalence. Post-hoc calculations suggested a minimum detectable effect of 19% relative reduction for *A*. *lumbricoides*, 41% for hookworm, and 52% for *T*. *trichiura*. Future studies in low-prevalence settings should enroll sample sizes large enough to detect small effects and use sensitive diagnostics.

We conducted multiple comparisons, increasing the risk of chance findings; the *T*. *trichiura* reduction in the sanitation but not WSH and N+WSH arms could indicate random error. However, most observed reductions followed consistent patterns that are unlikely to be explained by chance. Hookworm prevalence and intensity showed internally consistent reductions of similar size in all arms with a water or sanitation component, while the only intervention that achieved a borderline reduction in *A*. *lumbricoides* was combined WSH—the most biologically plausible intervention to reduce environmental transmission.

Another limitation is that we assessed STH outcomes 2.5 years after intervention initiation, which is a relatively short period of time to assess impact on *A*. *lumbricoides* given its long survival in soil [15]. This timeframe risks underestimating the long-term population benefit of reducing environmental soil contamination through improved sanitation. Longer-term follow-up of this population might provide a more accurate assessment of the long-term contribution of improved sanitation towards *A*. *lumbricoides* elimination.

Environmental conditions such as temperature, humidity and soil type affect the fate and transport of STH ova [40,46] and intervention effects are therefore likely to be setting-dependent. We controlled for month in our analysis to adjust for seasonality. Also, our geographically pair-matched randomization synchronized the timing of outcome measurement between arms, eliminating confounding from season as well as from unmeasured spatiotemporal factors. However, our findings may not be generalizable to other settings with different climatic and geological conditions, or different levels of fecal contamination in the ambient environment. Similarly, our findings are relevant to other populations with MDA programs and relatively low intensity of STH infection. Future studies should investigate the effect of water, sanitation and hygiene improvements on STH infection and how these can augment MDA programs in high-intensity infection settings.

### Conclusions

In a setting with ongoing MDA and low-intensity infections, we found modest but sustained reductions in hookworm prevalence and intensity from water treatment and combined WSH interventions. There was no STH reduction from handwashing and nutrition improvements. Intervention effects were more pronounced on hookworm than on *A*. *lumbricoides* and *T*. *trichiura;* this could be because of the short survival of hookworm in soil, precluding persistent environmental reservoirs of ova from pre-intervention contamination. Our findings suggest that drinking water can potentially be an overlooked transmission route for hookworm and that water treatment and sanitation interventions can augment MDA programs in striving towards breaking STH transmission.

## Supporting information

S1 Text Study protocol. (PDF)

S2 Text Pre-specified analysis plan. (PDF)

S3 Text CONSORT checklist. (PDF)

S4 Text Intervention promotion and user adherence. (PDF)

S5 Text Quality assurance for Kato-Katz. (PDF)

S6 Text Bias in effect estimates with imperfect sensitivity and specificity under non-differential classification. (PDF)

S1 Table Enrolment characteristics of individuals with missing vs. observed outcomes. (PDF)

S2 Table Infection prevalence, all interventions vs. control. (PDF)

S3 Table Infection prevalence, combined vs. individual WSH interventions. (PDF)

S4 Table Infection prevalence, combined N+WSH vs. WSH and nutrition interventions. (PDF)

S5 Table Moderate/heavy infection prevalence, all interventions vs. control. (PDF)

S6 Table Moderate/heavy infection prevalence, combined vs. individual WSH interventions. (PDF)

S7 Table Moderate/heavy infection prevalence, combined N+WSH vs. WSH and nutrition interventions. (PDF)

S8 Table Fecal egg count reduction, all interventions vs. control. (PDF)

S9 Table Fecal egg count reduction, combined vs. individual WSH interventions. (PDF)

S10 Table Fecal egg count reduction, combined N+WSH vs. WSH and nutrition interventions. (PDF)
