# Supplementary material for: Effects of water, sanitation, handwashing and nutritional interventions on soil-transmitted helminth infections in young children: A cluster-randomized controlled trial in rural Bangladesh
Source: PLoS Negl Trop Dis. 2019 May 3;13(5):e0007323. doi: 10.1371/journal.pntd.0007323 (PMC6519840; doi:10.1371/journal.pntd.0007323)
Supplement: S2 Text — (PDF) [file pntd.0007323.s002.pdf]

Text S2. Pre-specified analysis plan

The effects of single and combined water, sanitation, handwashing and nutritional interventions on soil transmitted helminth infections in young children: a cluster-randomized controlled trial in rural Bangladesh

Ayse Ercumen, Jade Benjamin-Chung, Benjamin F. Arnold, Audrie Lin, Alan Hubbard, Christine Stewart, Sarker Masud Parvez, Leanne Unicomb, Mahbubur Rahman, Rashidul Haque, John M. Colford Jr., Stephen P. Luby

Funding Source: Bill and Melinda Gates Foundation

Study Registration:
https://clinicaltrials.gov/ct2/show/study/NCT01590095?term=NCT01590095&rank=1

ClinicalTrials.gov Identifier: NCT01590095

Study Protocol:
Arnold BF, Null C, Luby SP, Unicomb L, Stewart CP, Dewey KG, et al. 2013. Cluster-randomised controlled trials of individual and combined water, sanitation, hygiene and nutritional interventions in rural Bangladesh and Kenya: the WASH Benefits study design and rationale. BMJ Open 3: e003476. (updated on 2016.02.05, https://osf.io/63mna/)

Date Updated: 19 January 2017

1. Scientific background and rationale

Soil transmitted helminth (STH) infections, including infections with *Ascaris lumbricoides* (roundworm), *Trichuris trichiura* (whipworm), and *Necator americanus* and *Ancylostoma duodenale* (hookworms) affect more than one billion people worldwide (Campbell et al. 2016). Chemotherapy in the form of mass drug administration with anthelmintic drugs such as benzimidazoles is effective in reducing infection (Keiser and Utzinger 2008; Vercruysse et al. 2011). However, the risk of developing drug resistance is a concern given the limited number of currently available drugs (Campbell et al. 2016). Additionally, in the absence of environmental measures to reduce transmission from feces to new hosts, rapid reinfection following treatment is common; a systematic review and meta-analysis has shown that, 12 months post-treatment, the infection prevalence reverts to 94% of pre-treatment levels for Ascaris, 82% for Trichuris and 57% for hookworm (Jia et al. 2012).

Improvements in water and sanitation infrastructure to break environmental transmission cycles of helminths have been identified as critical steps to sustainably reduce the global burden of STH infections (Anderson et al. 2014; Campbell et al. 2014). However, there is limited evidence to date on the impact of water, sanitation and hygiene (WSH) interventions on STH transmission (Campbell et al. 2016). Two recent systematic reviews found significantly reduced odds of STH infection associated with water treatment, sanitation access and handwashing (Strunz et al. 2014; Ziegelbauer et al. 2012); however, this evidence was mostly based on observational studies. There have been few randomized assessments of the effectiveness of WSH interventions in reducing STH transmission. Two

randomized controlled trials of sanitation interventions in rural India found no reduction in STH prevalence; these negative results could be because the intervention villages did not receive sufficiently high latrine coverage to break transmission cycles (Clasen et al. 2014; Patil et al. 2014). Randomized trials of hygiene and health education interventions have also had mixed success in reducing STH infections (Campbell et al. 2016). A randomized trial found that a school-based WSH program reduced reinfection prevalence for Ascaris but not for other STH species (Freeman et al. 2013). Similarly, the impact of host nutritional status on STH infections is poorly understood (Yap et al. 2014). WASH Benefits is the first randomized trial to assess the impact of individual and combined WSH and nutrition interventions on STH infection prevalence and intensity.

2. Objectives and hypotheses

The WASH Benefits study measures the impact of individual and combined WSH and nutrition interventions on growth, development and enteric disease in young children in Kenya and Bangladesh. This document outlines the STH outcomes collected in the Bangladesh trial, which aim to test the following hypotheses:

H1 (primary hypothesis): Do water, sanitation, handwashing and nutrition interventions, alone and in combination, reduce STH infection prevalence and intensity among young children?

H2: Do combined WSH interventions reduce STH infection prevalence and intensity among young children more than individual water, sanitation and handwashing interventions?

H3: Do combined WSH plus nutrition interventions reduce STH infection prevalence and intensity among young children more than WSH or nutrition alone?

3. Study design

The WASH Benefits trial has a cluster-randomized design, where eight households eligible for enrollment were defined to constitute a cluster and a buffer zone of a minimum of 15 minutes walking distance was enforced before enrolling the next cluster to minimize spillovers between enrolled clusters. Eight adjacent clusters were defined to form a block; clusters were randomized into study arms within the block, providing geographically pair-matched randomization to increase the efficiency of effect estimates as study outcomes are expected to be correlated with geographical characteristics. The trial had six intervention arms and a double-sized control arm.

4. Participants

The WASH Benefits Bangladesh trial was conducted in the Gazipur, Mymensingh, Tangail and Kishoreganj districts of central Bangladesh, selected based on the following criteria:

- Located in a rural area.
- Drinking water with low levels of iron (<1 mg/L on average) and arsenic (<50µg/L on average) as documented in the collaborative assessments by the Government of Bangladesh and the British Geological Survey. Water chemistry eligibility criteria were used because pilot studies indicated that when iron or arsenic levels were high the chlorine demand for household water treatment was unpredictable.
- The Government of Bangladesh, international nongovernment organizations working in Bangladesh and local government authorities report that no major water, sanitation or focused nutrition programmes are operating or planned in the area during the two-year study period.
- Not located in haor areas (areas completely submerged during the monsoon season).

In the selected districts, the trial enrolled compounds according to the following inclusion and exclusion criteria.

Inclusion criteria:
- Pregnant woman in her first or second trimester in the compound.
- The pregnant woman plans to stay in the village for the next 12 months.

Exclusion criteria:
- Household does not own their home (excluded to minimize loss to follow-up).
- Household draws water from a source with high iron content (excluded to optimize the effectiveness of the chlorine-based water treatment intervention).

The children born to the enrolled pregnant women were considered "index" children. STH outcomes were measured in the following children:

- Index children
- Children living in the enrolled compound that were aged 18-27 months at baseline
- One child aged 5-12 years per enrolled compound; we selected the youngest 5-12 year old sibling of the index child, if not available then the youngest 5-12 year old child living in the same household as the index child, if not available then the youngest 5-12 year old child living in the same compound as the index child

5. Intervention arms

The WASH Benefits intervention arms included water treatment, sanitation, handwashing, nutrition, combined water treatment, sanitation and handwashing (WSH) and combined WSH plus nutrition. The intervention packages entailed the following (Arnold et al. 2013).

- Water treatment: Chlorine tablets (Aquatabs; NaDCC) and safe storage vessel to treat and hygienically store drinking water.
- Sanitation: Child potties, sani-scoop hoes to remove feces from households and compounds, and latrine upgrades to a dual pit latrine for all households in study compounds.

- Handwashing: Handwashing stations near the latrine and kitchen, including soapy water bottles and detergent soap.
- Nutrition: Lipid-based nutrient supplements (LNS), exclusive breastfeeding and age-appropriate recommendations on maternal nutrition and infant feeding practices, in accordance with the Government of Bangladesh nutrition policy.

Local promoters visited study compounds at least twice a month to deliver behavior change messages that focused on (1) treating drinking water for children aged < 36 months, (2) use of latrines for defecation and disposal of human and animal feces from the compound, (3) handwashing with soap at critical times around food preparation, and after toilet use, contact with feces or cleaning child's anus, and (4) exclusive breastfeeding for index children aged 0-6 months, continued breastfeeding for index children through 24 months, use of LNS for index children aged 6-24 months and age-appropriate nutrition practices from pregnancy to 24 months of age for the index child.

The WSH interventions aimed to improve the environmental conditions of the index children from birth to reduce their early-life exposure to fecal pathogens. Households in Bangladesh are clustered in multiple-family compounds shared by members of extended families; the interventions aimed to modify the compound environment as the family compound was expected to present the primary domain of fecal exposure for young children, and compound-level environmental conditions have been shown to be associated with enteric outcomes in young children in rural Bangladesh (Lin et al. 2013).

6. Outcomes

STH outcomes were measured 24 months after intervention delivery, when index children were aged 30 months on average. Field staff distributed sterile stool collection containers to the primary caregivers of the children eligible for specimen collection, instructed them to collect stool from the following morning's defecation events, and returned to the household later on the day of defecation to retrieve the containers. Specimens were transported on ice to the field laboratory and analyzed on the same day for *Ascaris lumbricoides*, *Trichuris trichiura*, and *Necator americanus* and *Ancylostoma duodenale* (hookworm) ova using the double-slide Kato-Katz method (Katz et al. 1972). The Kato-Katz method provides quantitative measures of infection intensity, measured in eggs per gram (epg), as well as measures of infection prevalence. Infection intensity is associated with STH-related morbidity and transmission rates (Bethony et al. 2006; Brooker et al. 2006) and is therefore a recommended epidemiological measure to characterize STH infections and measure intervention impact (Anderson et al. 2014, 2013).

<u>Primary outcomes:</u>

- **Prevalence of STH infections** (Ascaris, Trichuris, hookworm), for organism-specific infection as well as infection with any organism.
- **Intensity of STH infections** (Ascaris, Trichuris, hookworm), measured in eggs per gram (epg) for organism-specific infection.

Secondary outcomes:

- **Intensity category of STH infections** (Ascaris, Trichuris, hookworm), measured as low, moderate or high intensity infection using the following WHO cut-offs based on eggs per gram of stool (WHO 2002).

	Low intensity (epg)	Moderate intensity (epg)	High intensity (epg)
Ascaris	1-5000	5000-50000	>50000
Trichuris	1-1000	1000-10000	>10000
Hookworm	1-2000	2000-4000	>4000

7. Minimum detectable effect size

The WASH Benefits study was designed and sized to detect intervention impacts on child length and diarrhea. The selected sample size for the study was 5,040 pregnant mothers in 720 clusters (7 mothers/cluster). To calculate the minimum detectable effect (MDE) for this sample size, we assumed: (1) Two children per enrolled household eligible for stool collection, and (2) 70% stool recovery. We estimated the infection prevalence and intraclass correlation coefficient (ICC) in the control group using data from a study in rural Bangladesh that measured STH outcomes in ~1000 preschool-aged and school-aged children (Benjamin-Chung et al. 2015). With these assumptions, the selected sample size allows the following MDEs between any intervention arm and the double-sized control arm with 80% power and a two-sided α of 0.05. We note that, with the given sample size and this set of assumptions, the study is powered to detect only relatively large reductions in the prevalence of individual STH species; however, these MDEs are subject to change depending on the actual prevalence and ICC values that we observe in our study.

	Prevalence in control arm (%)	ICC	Relative Reduction (%)
Ascaris	14	0.11	41
Trichuris	22	0.21	39
Hookworm	5	0.02	50
Any STH	50	0.14	18

8. Effect modifiers

Age is strongly correlated with the intensity and prevalence of STH infections and different species display different patterns of age-dependence. Ascaris and Trichuris infection intensity peaks in children aged 5-15 years and declines in adults, while hookworm infection intensity increases with age (Anderson et al. 2014). Infection prevalence for all three species peaks in school-aged children and remains at a plateau into adulthood (Anderson et al. 2014). The reasons for these age-dependent patterns include temporal changes in exposure, changes in immunity with increasing age or both (Galvani 2005).

Other factors that potentially affect STH outcomes include the number of individuals and the number of school-aged children living in the compound, recent deworming (Benjamin-Chung et al. 2015), floor material (Benjamin-Chung et al. 2015), wearing shoes (Tomczyk et al. 2014) and geophagia (Glickman et al. 1999). Additionally, baseline sanitary conditions could impact the magnitude of the reductions in STH infections achieved by the WASH Benefits interventions; compounds with the poorest sanitary conditions at the time of enrollment would presumably experience greater benefits from the interventions.

Finally, the sensitivity of the Kato-Katz method has been shown to decline with increasing time gap between defecation and sample processing (Dacombe et al. 2007). We targeted to collect stool samples from defecation events that occurred on the day of sample processing; however, a subset of participants submitted samples from the previous day's defecation events. These older samples could have lower ova counts due to reduced sensitivity.

9. Analysis

9.1 Statistical parameters

To test our hypotheses, we will conduct comparisons between the following study arms.

H1 (primary hypothesis): Compare STH infection prevalence and intensity in the water, sanitation, handwashing, combined WSH, nutrition and combined WSH plus nutrition arms to the double-sized control arm.

H2: Compare STH infection prevalence and intensity in the combined WSH arm to the individual water, sanitation and handwashing arms.

H3: Compare STH infection prevalence and intensity in the combined WSH plus nutrition arm to the WSH and nutrition arms.

We will estimate unadjusted and adjusted intention-to-treat effects between study arms; the intention-to-treat parameter preserves the benefits of the randomized design and is an appropriate measure of intervention effectiveness given the high user uptake of the WASH Benefits interventions (Luby et al. *in prep*). Our parameters of interest for infection prevalence will be the prevalence differences and ratios. Our parameter of interest for helminth intensity will be the relative reduction in infection intensity. We will calculate the relative reduction using both geometric and arithmetic means (Vercruysse et al. 2011). We will estimate these same parameters in the subgroup analyses specified in section 9.3 below.

9.2 Unadjusted and adjusted analyses

Randomization led to extremely good balance in measured covariates across arms (Luby et al. *in prep*), and bias due to confounding in the trial is extremely unlikely. Therefore we will rely on the unadjusted analysis as our primary analysis. We will estimate the unadjusted prevalence ratio, prevalence difference and relative reduction infection intensity parameters using targeted maximum likelihood estimation (TMLE). TMLE is implemented in the tmle package in the R statistical software (Gruber and van der Laan 2011).

In a secondary analysis, we will estimate adjusted parameters by including variables that are strongly associated with the outcome to potentially improve the precision of our estimates (decrease the SEs); however, we recognize that for binary outcomes gains in precision are very unlikely (Pocock et al. 2002). In accordance with the main study's analysis plan (Arnold et al. 2013) (updated on 2016.02.05, https://osf.io/63mna/), we will consider and test the following covariates:

- ID of the lab staff member who performed the Kato-Katz analysis
- Month of measurement, to account for seasonal variation
- Child age (days)
- Child sex
- Child birth order
- Mother's age (years)
- Mother's height (cm)
- Mother's education level (no education, primary, secondary)
- Household food insecurity (4-level HFIAS categories)
- Number of children < 18 years in the household
- Number of individuals living in the compound
- Distance (in minutes) to the household's primary drinking water source
- Housing materials (floor, walls, roof) and household assets
  - Assets measured: electricity, wardrobe, table, chair or bench, watch or clock, khat, chouki, working radio, working black/white or color television, refrigerator, bicycle (not child's toy), motorcycle, sewing machine, mobile phone, land phone

As specified in the original protocol, we will conduct the adjusted analyses using TMLE. TMLE enables use of the SuperLearner ensemble machine learning algorithm to flexibly adjust for pre-specified covariates (Laan et al. 2007). We will include the following model library in the ensemble (SuperLearner R package arguments in parenthesis): the simple mean (SL.mean), main effects generalized linear models (SL.glm), Bayesian generalized linear models (SL.bayesglm) (Gelman et al. 2008), generalized additive models degree 2 (SL.gam) (Hastie and Tibshirani 1990), and penalized elastic net regression (SL.glmnet) (Friedman et al. 2010).

We will pre-screen covariates to assess whether they are associated with each outcome prior to including them in adjusted statistical models (Pocock et al. 2002). We will use the likelihood ratio test to assess the association between each outcome and each covariate and will include covariates with a p-value < 0.2 in the adjusted analysis. We will also exclude covariates that have little variation in the study population (e.g., prevalence <5%).

9.3 Effect modification and subgroup analyses

We will tabulate infection intensity and prevalence by the following covariates as well as conduct subgroup analyses by these covariates by estimating our parameters of interest within the corresponding subsets of the dataset.

- Index children (who were the primary recipients of the interventions)
- Pre-school-aged (2-4 yrs) vs. school-aged (5-12 yrs) children, consistent with previously used age groupings (Anderson et al. 2014)
- Number of individuals living in the compound (<10 vs. ≥10)
- Percentage of mud floors in household and latrine area (0 vs. ≥1)
- Caregiver-reported deworming of child in last six months
- Samples from same-day vs. previous-day defecation
- At baseline, compound had latrine with water seal that flushes into pit or septic tank
- At baseline, compound had dedicated tool to clean up feces and reports using it to dispose of child feces
- At baseline, no open defecation was reported by any compound member

The binary cut-offs for categorical effect modifiers (number of individuals and school-aged children living in compound, and percentage of mud floors in the household and latrine areas) were determined based on the empirical data distribution for these variables. We note that the selected sample size is not powered to detect interactions – these subgroup analyses should be interpreted as pre-specified exploratory analyses.

9.4 Missing specimens

We will tabulate the percentage of participants lost to follow-up between enrollment and specimen collection at the two-year follow-up as well as the percentage of participants that completed the study but from whom a stool specimen was not successfully obtained. We will assess whether missing specimens are differential with respect to treatment group or any characteristics of participants, by comparing the percentage of missingness across study arms and comparing baseline covariates between those with an available vs. missing specimen (Wood et al. 2004). We will also compare the balance of baseline covariates between study arms both in the original enrolled population and among participants that successfully provided a specimen. We will conduct a complete-case analysis, and if we find evidence of differential missingness by study arm or by any covariates that predict the outcome, we will also conduct an inverse probability of censoring-weighted (IPCW) analysis that reconstructs the original enrolled population (Hernán et al. 2004).

Individuals with missing specimens will be imputed as follows: For all households that were enrolled at baseline and had a subsequent index child live birth but were then lost to follow-up by the time of the STH assessment, an index child will be imputed. For a random subset of these households, an older non-index child will also be imputed based on what percent of non-lost households had an eligible (5-12 yrs old) non-index child. Among households that remained enrolled at the time of the STH assessment, all individuals with a missing specimen will be imputed.

The IPCW analysis will screen and adjust for differential missingness by the same adjustment covariates listed in 9.2 except for ID of the lab staff member who performed the Kato-Katz analysis and month of Kato-Katz analysis as well as child age, sex and birth order since this information is not available for children in households that were lost after baseline (index children were not born at the time of baseline data collection and this

information was therefore collected at the time of the one-year assessment for the trial's main outcomes). Instead, an indicator variable distinguishing index vs. non-index child status will be included as an adjustment covariate to serve as a proxy for age for imputed children. This variable captures differential missingness by child age (older non-index children were more likely to have a missing specimen than index children) as well as age-dependent variation in STH outcomes (older non-index children had higher STH prevalence than index children but STH prevalence did not vary significantly with age among index children and among non-index children). The IPCW analysis will be implemented using TMLE. We will estimate inverse probability of censoring weights as a function of the specified covariates using data-adaptive super learning and adjust the effect estimates for missingness.

Post-Hoc Changes to Analysis Plan

(1) Switch from Mantel-Haenszel estimator to TMLE for unadjusted parasite prevalence outcomes. The analysis plan had originally proposed using the Mantel-Haenszel (MH) estimator for the binary STH outcomes to account for the pair-matching in the study design. However, upon completing these pre-specified analyses, we discovered that for the rare STH outcomes such as hookworm and Trichuris, the MH estimator drops a substantial number of blocks (as many as 50%) in which there are no outcomes in a given arm -- the MH estimator drops strata with no events because it cannot estimate a PR within those strata. This discards a lot of information and the MH estimator is consequently driven by a small subset of the available observations. We therefore revised our approach to use TMLE for the unadjusted STH prevalence analyses.

(2) Remove the subgroup analysis by the number of school-aged children in the compound. Since school-aged non-index children have higher STH prevalence than pre-school aged index children and many compounds only have one school-aged child, the higher prevalence in compounds with a school-aged child is primarily driven by the prevalence in these children themselves (in which case this subgroup analysis does not provide any additional information compared to the one on index vs. older non-index children).

(3) Remove the subgroup analyses by geophagia and wearing shoes. Since these variables are strongly age-dependent and age is a strong predictor of STH prevalence, we cannot tease out the age effect from the effect of these.

(4) Remove formal tests of effect modification. An examination of overlapping confidence intervals among subgroups demonstrates that there is no significant effect modification.

References

Anderson R, Truscott J, Hollingsworth TD. 2014. The coverage and frequency of mass drug administration required to eliminate persistent transmission of soil-transmitted helminths. Phil Trans R Soc B 369:20130435; doi:10.1098/rstb.2013.0435.

Anderson RM, Truscott JE, Pullan RL, Brooker SJ, Hollingsworth TD. 2013. How Effective Is School-Based Deworming for the Community-Wide Control of Soil-Transmitted Helminths? P.J. Lammieed. PLoS Negl. Trop. Dis. 7:e2027; doi:10.1371/journal.pntd.0002027.

Arnold BF, Null C, Luby SP, Unicomb L, Stewart CP, Dewey KG, et al. 2013. Cluster-randomised controlled trials of individual and combined water, sanitation, hygiene and nutritional interventions in rural Bangladesh and Kenya: the WASH Benefits study design and rationale. BMJ Open 3: e003476.

Benjamin-Chung J, Nazneen A, Halder AK, Haque R, Siddique A, Uddin MS, et al. 2015. The Interaction of Deworming, Improved Sanitation, and Household Flooring with Soil-Transmitted Helminth Infection in Rural Bangladesh. PLOS Negl Trop Dis 9:e0004256; doi:10.1371/journal.pntd.0004256.

Bethony J, Brooker S, Albonico M, Geiger SM, Loukas A, Diemert D, et al. 2006. Soil-transmitted helminth infections: ascariasis, trichuriasis, and hookworm. The Lancet 367:1521–1532; doi:10.1016/S0140-6736(06)68653-4.

Brooker S, Clements ACA, Bundy DAP. 2006. Global Epidemiology, Ecology and Control of Soil-Transmitted Helminth Infections. In *Advances in Parasitology*, Vol. 62 of, pp. 221–261, Elsevier.

Campbell SJ, Nery SV, McCarthy JS, Gray DJ, Soares Magalhães RJ, Clements ACA. 2016. A Critical Appraisal of Control Strategies for Soil-Transmitted Helminths. Trends Parasitol. 32:97–107; doi:10.1016/j.pt.2015.10.006.

Campbell SJ, Savage GB, Gray DJ, Atkinson J-AM, Magalhães RJS, Nery SV, et al. 2014. Water, Sanitation, and Hygiene (WASH): A Critical Component for Sustainable Soil-Transmitted Helminth and Schistosomiasis Control. PLOS Negl Trop Dis 8:e2651; doi:10.1371/journal.pntd.0002651.

Clasen T, Boisson S, Routray P, Torondel B, Bell M, Cumming O, et al. 2014. Effectiveness of a rural sanitation programme on diarrhoea, soil-transmitted helminth infection, and child malnutrition in Odisha, India: a cluster-randomised trial. Lancet Glob. Health 2: e645–e653.

Dacombe RJ, Crampin AC, Floyd S, Randall A, Ndhlovu R, Bickle Q, et al. 2007. Time delays between patient and laboratory selectively affect accuracy of helminth diagnosis. Trans. R. Soc. Trop. Med. Hyg. 101:140–145; doi:10.1016/j.trstmh.2006.04.008.

Freeman MC, Clasen T, Brooker SJ, Akoko DO, Rheingans R. 2013. The Impact of a School-Based Hygiene, Water Quality and Sanitation Intervention on Soil-Transmitted Helminth Reinfection: A Cluster-Randomized Trial. Am. J. Trop. Med. Hyg. 89:875–883; doi:10.4269/ajtmh.13-0237.

Friedman J, Hastie T, Tibshirani R. 2010. Regularization Paths for Generalized Linear Models via Coordinate Descent. J. Stat. Softw. 33: 1–22.

Galvani AP. 2005. Age-Dependent Epidemiological Patterns and Strain Diversity in Helminth Parasites. J. Parasitol. 91: 24–30.

Gelman A, Jakulin A, Pittau MG, Su Y-S. 2008. A Weakly Informative Default Prior Distribution for Logistic and Other Regression Models. Ann. Appl. Stat. 2: 1360–1383.

Glickman LT, Camara AO, Glickman NW, McCabe GP. 1999. Nematode intestinal parasites of children in rural Guinea, Africa: prevalence and relationship to geophagia. Int. J. Epidemiol. 28:169–174; doi:10.1093/ije/28.1.169.

Gruber S, van der Laan MJ. 2011. tmle: An R package for targeted maximum likelihood estimation.

Hastie TJ, Tibshirani RJ. 1990. *Generalized Additive Models*. CRC Press.

Hernán MA, Hernández-Díaz S, Robins JM. 2004. A Structural Approach to Selection Bias: Epidemiology 15:615–625; doi:10.1097/01.ede.0000135174.63482.43.

Jia T-W, Melville S, Utzinger J, King CH, Zhou X-N. 2012. Soil-Transmitted Helminth Reinfection after Drug Treatment: A Systematic Review and Meta-Analysis. PLOS Negl Trop Dis 6:e1621; doi:10.1371/journal.pntd.0001621.

Katz N, Chaves A, Pellegrino J. 1972. A simple device for quantitative stool thick-smear technique in Schistosomiasis mansoni. Rev. Inst. Med. Trop. Sao Paulo 14: 397–400.

Keiser J, Utzinger J. 2008. Efficacy of current drugs against soil-transmitted helminth infections: Systematic review and meta-analysis. JAMA 299:1937–1948; doi:10.1001/jama.299.16.1937.

Laan VD, J M, Polley EC, Hubbard AE. 2007. Super Learner. Stat. Appl. Genet. Mol. Biol. 6.

Lin A, Arnold BF, Afreen S, Goto R, Huda TMN, Haque R, et al. 2013. Household Environmental Conditions Are Associated with Enteropathy and Impaired Growth in Rural Bangladesh. Am. J. Trop. Med. Hyg. 89:130–137; doi:10.4269/ajtmh.12-0629.

Patil SR, Arnold BF, Salvatore AL, Briceno B, Ganguly S, Jr JMC, et al. 2014. The Effect of India's Total Sanitation Campaign on Defecation Behaviors and Child Health in Rural

Madhya Pradesh: A Cluster Randomized Controlled Trial. PLOS Med 11:e1001709; doi:10.1371/journal.pmed.1001709.

Pocock SJ, Assmann SE, Enos LE, Kasten LE. 2002. Subgroup analysis, covariate adjustment and baseline comparisons in clinical trial reporting: current practiceand problems. Stat. Med. 21:2917–2930; doi:10.1002/sim.1296.

Strunz EC, Addiss DG, Stocks ME, Ogden S, Utzinger J, Freeman MC. 2014. Water, Sanitation, Hygiene, and Soil-Transmitted Helminth Infection: A Systematic Review and Meta-Analysis. PLOS Med 11:e1001620; doi:10.1371/journal.pmed.1001620.

Tomczyk S, Deribe K, Brooker SJ, Clark H, Rafique K, Knopp S, et al. 2014. Association between Footwear Use and Neglected Tropical Diseases: A Systematic Review and Meta-Analysis. PLOS Negl Trop Dis 8:e3285; doi:10.1371/journal.pntd.0003285.

VanderWeele TJ, Knol MJ. 2014. A Tutorial on Interaction. Epidemiol. Methods 3; doi:10.1515/em-2013-0005.

Vercruysse J, Behnke JM, Albonico M, Ame SM, Angebault C, Bethony JM, et al. 2011. Assessment of the Anthelmintic Efficacy of Albendazole in School Children in Seven Countries Where Soil-Transmitted Helminths Are Endemic. PLOS Negl Trop Dis 5:e948; doi:10.1371/journal.pntd.0000948.

WHO. 2002. Prevention and control of schistosomiasis and soil-transmitted helminthiasis. World Health Organ. Tech. Rep. Ser. 912: i–vi, 1-57, back cover.

Wood AM, White IR, Thompson SG. 2004. Are missing outcome data adequately handled? A review of published randomized controlled trials in major medical journals. Clin. Trials 1:368–376; doi:10.1191/1740774504cn032oa.

Yap P, Utzinger J, Hattendorf J, Steinmann P. 2014. Influence of nutrition on infection and re-infection with soil-transmitted helminths: a systematic.

Ziegelbauer K, Speich B, Mäusezahl D, Bos R, Keiser J, Utzinger J. 2012. Effect of Sanitation on Soil-Transmitted Helminth Infection: Systematic Review and Meta-Analysis. PLoS Med. 9: e1001162.
