# Supplementary material for: Effects of water, sanitation, handwashing and nutritional interventions on soil-transmitted helminth infections in young children: A cluster-randomized controlled trial in rural Bangladesh
Source: PLoS Negl Trop Dis. 2019 May 3;13(5):e0007323. doi: 10.1371/journal.pntd.0007323 (PMC6519840; doi:10.1371/journal.pntd.0007323)
Supplement: S3 Text — (PDF) [file pntd.0007323.s003.pdf]

Text S3. CONSORT checklist

Item	Description	Reported in Section
**Title and Abstract**		
1a	Identification as a randomized trial in the title; Identification as a cluster randomized trial in the title	Abstract
1b	Structured summary of trial design, methods, results, and conclusions	Abstract
**Introduction**		
Background and Objectives		
2a	Scientific background and explanation of rationale; Rationale for using a cluster design	Introduction
2b	Specific objectives or hypotheses; Whether objectives pertain to the cluster level, the individual participant level, or both	Introduction
**Methods**		
Trial Design		
3a	Description of trial design (such as parallel, factorial) including allocation ratio; Definition of cluster and description of how the design features apply to the clusters	Methods (Randomization and masking section)
3b	Important changes to methods after trial commencement (such as eligibility criteria), with reasons	N/A
Participants		
4a	Eligibility criteria for participants; Eligibility criteria for clusters	Methods (Randomization and masking section)
4b	Settings and locations where the data were collected	Methods (Study setting section)
Interventions		
5	The interventions for each group with sufficient details to allow replication, including how and when they were actually administered; Whether interventions pertain to the cluster level, the individual participant level, or both	Methods (Interventions section)
Outcomes		
6a	Completely defined pre-specified primary and secondary outcome measures, including how and when they were assessed; Whether outcome measures pertain to the cluster level, the individual participant level, or both	Methods (Outcome assessment section)

Item	Description	Reported in Section
6b	Any changes to trial outcomes after the trial commenced, with reasons	N/A
Sample Size		
7a	How sample size was determined; Method of calculation, number of cluster(s) (and whether equal or unequal cluster sizes are assumed), cluster size, a coefficient of intracluster correlation (ICC or k), and an indication of its uncertainty	Methods (Statistical analysis section)
7b	When applicable, explanation of any interim analyses and stopping guidelines	N/A
**Randomization**		
Sequence Generation		
8a	Method used to generate the random allocation sequence	Methods (Randomization and masking section)
8b	Type of randomization; details of any restriction (such as blocking and block size); Details of stratification or matching if used	Methods (Randomization and masking section)
Allocation Concealment Mechanism		
9	Mechanism used to implement the random allocation sequence (such as sequentially numbered containers), describing any steps taken to conceal the sequence until interventions were assigned; Specification that allocation was based on clusters rather than individuals and whether allocation concealment (if any) was at the cluster level, the individual participant level, or both	Methods (Randomization and masking section)
Implementation		
10a	Who generated the random allocation sequence, who enrolled clusters, and who assigned clusters to interventions	Methods (Randomization and masking section)
10b	Mechanism by which individual participants were included in clusters for the purposes of the trial (such as complete enumeration, random sampling)	Methods (Randomization and masking section)
10c	From whom consent was sought (representatives of the cluster, or individual cluster members, or both) and whether consent was sought before or after randomization	Methods (Ethics section)
Blinding		
11a	If done, who was blinded after assignment to interventions (for example, participants, care providers, those assessing outcomes) and how	Methods (Randomization and masking section)

Item	Description	Reported in Section
11b	If relevant, description of the similarity of interventions	N/A
Statistical Methods		
12a	Statistical methods used to compare groups for primary and secondary outcomes; How clustering was taken into account	Methods (Statistical analysis section)
12b	Methods for additional analyses, such as subgroup analyses and adjusted analyses	Methods (Statistical analyses section)
**Results**		
Participant Flow		
13a	For each group, the numbers of participants/clusters who were randomly assigned, received intended treatment, and were analyzed for the primary outcome	Results (Enrollment section), Figure 1 (CONSORT diagram)
13b	For each group, losses and exclusions after randomization, together with reasons, for both clusters and individual cluster members	Results (Enrollment section), Figure 1 (CONSORT diagram)
Recruitment		
14a	Dates defining the periods of recruitment and follow-up	Results (Enrollment section)
14b	Why the trial ended or was stopped	N/A
Baseline Data		
15	A table showing baseline demographic and clinical characteristics for each group; Baseline characteristics for the individual and cluster levels as applicable for each group	Table 1
Numbers Analysed		
16	For each group, number of participants/clusters (denominator) included in each analysis and whether the analysis was by the original assigned groups	Methods (Statistical analyses section), Figure 3, Figure 4
Outcomes and Estimation		
17a	For each primary and secondary outcome, results for each group, and the estimated effect size and its precision (such as 95% confidence interval); Results at the individual and cluster levels as applicable and a coefficient of intracluster correlation (ICC or k) for each primary outcome	Results (Interventions vs. control section, Combined vs. single interventions section), Figure 3, Figure 4
17b	For binary outcome, presentation of both absolute and relative effect sizes is recommended	Results (Interventions vs. control section, Combined vs. single interventions section), Figure 3, Figure 4

Item	Description	Reported in Section
Ancillary Analyses		
18	Results of any other analyses performed, including subgroup analyses and adjusted analyses, distinguishing pre-specified from exploratory	Results (Other effects section, Subgroup analyses section)
Harms		
19	All important harms or unintended effects in each group	N/A
**Discussion**		
Limitations		
20	Trial limitations, addressing sources of potential bias, imprecision and, if relevant, multiplicity of analyses	Discussion (Limitations section)
Generalisability		
21	Generalisability (external validity, applicability) of the trial findings; Generalisability to clusters and/or individual participants (as relevant)	Discussion (Limitations section)
Interpretation		
22	Interpretation consistent with results, balancing benefits and harms, and considering other relevant evidence	Discussion
**Other Information**		
Registration		
23	Registration number and name of trial registry	Methods (Registration section)
Protocol		
24	Where the full trial protocol can be accessed, if available	Methods (Registration section)
Funding		
25	Sources of funding and other support (such as supply of drugs), role of funders	Acknowledgements
