# Supplementary material for: Effects of water, sanitation, handwashing and nutritional interventions on soil-transmitted helminth infections in young children: A cluster-randomized controlled trial in rural Bangladesh
Source: PLoS Negl Trop Dis. 2019 May 3;13(5):e0007323. doi: 10.1371/journal.pntd.0007323 (PMC6519840; doi:10.1371/journal.pntd.0007323)
Supplement: S4 Text — (PDF) [file pntd.0007323.s004.pdf]

Text S4. Intervention promotion and user adherence

WASH Benefits included an extensive promotion effort to ensure user adherence with the interventions. The trial hired and trained local women to serve as community health promoters; these were hired specifically for WASH Benefits and were not involved in other ongoing programs (e.g., MDA) in the study area. Community health promoters were instructed to visit participants in intervention arms in their home at least bi-weekly throughout the trial and visited households six times per month on average. During these visits, they delivered and replenished the supply of intervention products, demonstrated their correct use, resolved hardware problems and encouraged their consistent use through discussion with participants. The targeted intervention adherence behaviors were: (1) children <36 months drinking chlorinated and safely stored water, (2) use of latrines for defecation and disposal of human and animal feces, (3) handwashing with soap at specified critical times (before food preparation, and after defecation and contact with feces) and (4) consumption of provided nutritional supplements and adoption of the promoted age-appropriate feeding behaviors. Control arm participants were not visited by community health promoters.

Intervention adherence was measured throughout the trial with multiple methods. Field staff conducted (1) unannounced spot check observations and a structured questionnaire in a random subset of 224 households per month over the course of 20 months starting after approximately two months of intervention, and (2) structured observations in a random subset of 324 households after approximately 15 months of intervention. The spot check observations assessed whether the provided intervention products were available, functional and had visible signs of use while the structured questionnaire recorded reported behaviors (e.g., water treatment, latrine use), and the structured observations included direct monitoring of the promoted behaviors. Stored water samples were tested for residual free chlorine in households reporting chlorine water treatment to validate the self-reported practices. Further details of the adherence assessment methods are described elsewhere [1].

All assessments showed high intervention adherence, both in terms of the observed status of the hardware and observed practices. In all arms with a sanitation component, 95-98% of households had a latrine with a functional water seal compared to 23% of controls [1]. Stool was observed on the latrine slab/floor or immediately outside the latrine in 24-38% of sanitation households vs. 62% of control households [1]. During structured observations, 94-97% of adults in sanitation arms were observed to defecate in a hygienic latrine compared to 40% of adults in the control arm [1]. In contrast, child defecation practices were similar between arms; in sanitation arms, 37-54% of young children were observed to defecate in a latrine or potty vs. 32% of control children [1]. In arms with a handwashing component, 77-85% of households had observed water and soap at the latrine and food preparation areas compared to 21% of controls [1]. During structured observations, 67-74% of participants in handwashing arms washed hands after latrine use vs. 29% of controls [1]. However, handwashing before food handling was infrequent (<10%) in all arms [1]. In all arms with a water treatment component, 50-70% of households had detectable residual free chlorine in their stored water; no household had chlorinated water in the control arm [1]. In 56-59% of households with the nutrition

intervention, caregivers were observed to feed ≥1 nutritional supplement package to index children during five hours of structured observation [1]. Further details of adherence are described elsewhere [1].

1. Parvez SM, Azad R, Rahman M, Unicomb L, Ram PK, Naser AM, et al. Achieving optimal technology and behavioral uptake of single and combined interventions of water, sanitation hygiene and nutrition, in an efficacy trial (WASH benefits) in rural Bangladesh. Trials. 2018;19. doi:10.1186/s13063-018-2710-8
