# Supplementary material for: Effects of water, sanitation, handwashing and nutritional interventions on soil-transmitted helminth infections in young children: A cluster-randomized controlled trial in rural Bangladesh
Source: PLoS Negl Trop Dis. 2019 May 3;13(5):e0007323. doi: 10.1371/journal.pntd.0007323 (PMC6519840; doi:10.1371/journal.pntd.0007323)
Supplement: S5 Text — (PDF) [file pntd.0007323.s005.pdf]

**Text S5. Quality assurance for Kato-Katz**

A team of four laboratory technicians was trained at the parasitology laboratory of the International Centre for Diarrhoeal Disease Research, Bangladesh (icddr,b) for STH enumeration by Kato-Katz. 10% of Kato-Katz slides were counted by two technicians, and 5% were counted by a senior parasitologist for quality assurance. We assessed interrater agreement between two individual technicians as well as between a given technician and the senior parasitologist by calculating the kappa statistic for slides categorized as positive [1].

The kappa statistic for interrater agreement between two laboratory technicians was 1.00 for *A. lumbricoides* and 0.99 for hookworm and *T. trichiura*. The average kappa statistic for agreement between the laboratory technician that performed the original count and the experienced parasitologist was 0.92 for *A. lumbricoides*, 0.20 for hookworm, and 0.86 for *T. trichiura*. For hookworm, the kappa statistic decreased with the number of days since the slide had been prepared and the original count had been conducted at the field laboratory; the kappa statistic was 1.00 for samples where the experienced parasitologist counted the slides on the day of the original count, 0.33 for samples counted one day later, and 0.11 for samples counted 2-4 days later.

<u>Technician 1 vs. 2</u>

| ***A. lumbricoides*** | Not detected by technician 2 | Detected by technician 2 | Total |
|---|---|---|---|
| Not detected by technician 1 | 850 | 2 | 852 |
| Detected by technician 1 | 1 | 939 | 940 |
| Total | 851 | 941 | 1792 |

Kappa = 0.997

| **Hookworm** | Not detected by technician 2 | Detected by technician 2 | Total |
|---|---|---|---|
| Not detected by technician 1 | 1652 | 0 | 1652 |
| Detected by technician 1 | 2 | 138 | 140 |
| Total | 1654 | 138 | 1792 |

Kappa = 0.992

| ***T. trichiura*** | Not detected by technician 2 | Detected by technician 2 | Total |
|---|---|---|---|
| Not detected by technician 1 | 1645 | 1 | 1646 |
| Detected by technician 1 | 1 | 147 | 148 |
| Total | 1646 | 148 | 1794 |

Kappa = 0.993

Technician vs. senior parasitologist

| ***A. lumbricoides*** | Not detected by senior | Detected by senior | Total |
|---|---|---|---|
| Not detected by technician | 405 | 5 | 410 |
| Detected by technician | 25 | 291 | 316 |
| Total | 430 | 296 | 726 |

Kappa = 0.915

| **Hookworm** | Not detected by senior | Detected by senior | Total |
|---|---|---|---|
| Not detected by technician | 689 | 0 | 689 |
| Detected by technician | 30 | 4 | 34 |
| Total | 719 | 4 | 723 |

Kappa = 0.203

| ***T. trichiura*** | Not detected by senior | Detected by senior | Total |
|---|---|---|---|
| Not detected by technician | 664 | 2 | 666 |
| Detected by technician | 12 | 46 | 58 |
| Total | 676 | 48 | 724 |

Kappa = 0.858

1. McHugh ML. Interrater reliability: the kappa statistic. Biochemia medica : Biochemia medica. 2012;22: 276–282.
