# Supplementary material for: Effects of water, sanitation, handwashing and nutritional interventions on soil-transmitted helminth infections in young children: A cluster-randomized controlled trial in rural Bangladesh
Source: PLoS Negl Trop Dis. 2019 May 3;13(5):e0007323. doi: 10.1371/journal.pntd.0007323 (PMC6519840; doi:10.1371/journal.pntd.0007323)
Supplement: S6 Text — (PDF) [file pntd.0007323.s006.pdf]

Text S6. Bias in effect estimates with imperfect sensitivity and specificity under non-differential classification

<u>Definitions</u>:

P1	True probability of disease in intervention group
P0	True probability of disease in control group
P1*	Measured probability of disease in intervention group
P0*	Measured probability of disease in control group
PR	True prevalence ratio between intervention vs. control (P1/P0)
PD	True prevalence difference between intervention vs. control (P1-P0)
PR*	Measured prevalence ratio between intervention vs. control (P1*/P0*)
PD*	Measured prevalence difference between intervention vs. control (P1*-P0*)
x1	Probability of false negatives in intervention group
x0	Probability of false negatives in control group
y1	Probability of false positives in intervention group
y0	Probability of false positives in control group

$P1^* = P1 - P1\,x1 + (1-P1)\,y1$

$P0^* = P0 - P0\,x0 + (1-P0)\,y0$

<u>Assumptions under non-differential misclassfication:</u>

$x1 = x0 = x$

$y1 = y0 = y$

$P1^* = P1 - P1\,x + (1-P1)\,y$

$P0^* = P0 - P0\,x + (1-P0)\,y$

<u>Scenario 1:</u> Imperfect sensitivity, perfect specificity ($x > 0$, $y = 0$)

$$PR^* = \frac{P1 - P1\,x}{P0 - P0\,x} = \frac{P1\,(1 - x)}{P0\,(1 - x)} = \frac{P1}{P0} = PR$$

$PD^* = P1 - P1\,x - (P0 - P0\,x) = P1\,(1 - x) - P0\,(1 - x) = (1 - x)\,(P1 - P0) = (1 - x)\,PD$

Scenario 2: Perfect sensitivity, imperfect specificity ($x = 0, y > 0$)

$$PR^* = \frac{P1 + (1 - P1)\,y}{P0 + (1 - P0)\,y} = \frac{P1 + y - P1\,Y}{P0 + y - P0\,y} = \frac{P1\,(1 - y + y/P1)}{P0\,(1 - y + y/p0)} = \frac{P1}{P0} \cdot \frac{1 - y + y/P1}{1 - y + y/P0} = PR \cdot \frac{1 - y + y/P1}{1 - y + y/P0}$$

when $P1 < P0$, $PR^* > PR$
when $P1 > P0$, $PR^* < PR$

$$PD^* = P1 + (1 - P1)\,y - [\,P0 + (1 - P0)\,y\,] = P1 + y - P1\,y - (P0 + y - P0y\,) = P1\,(1 - y) - P0\,(1 - y) = (1 - y)\,(P1 - P0) = (1-y)\,PD$$

Scenario 3: Imperfect sensitivity, imperfect sensitivity ($x > 0, y > 0$)

$$PR^* = \frac{P1 - P1\,x + (1 - P1)\,y}{P0 - P0\,x + (1 - P0)\,y} = \frac{P1 - P1\,x + y - P1\,y}{P0 - P0\,x + y - P0\,y} = \frac{P1\,(1 - x - y + y/P1)}{P0\,(1 - x - y + y/P0)} = \frac{P1}{P0} \cdot \frac{1 - x - y + y/P1}{1 - x - y + y/P0} = PR \cdot \frac{1 - x - y + y/P1}{1 - x - y + y/P0}$$

when $P1 < P0$, $PR^* > PR$
when $P1 > P0$, $PR^* < PR$

$$PD^* = P1 - P1\,x + (1 - P1)\,y - [\,P0 - P0\,x + (1 - P0)\,y\,] = P1 - P1\,x + y - P1\,y - (P0 - P0\,x + y - P0\,y) = P1\,(1 - x - y\,) - P0\,(1 - x - y) = (1 - x - y\,)\,(P1 - P0) = (1 - x - y)\,PD$$
