# Supplementary material for: Effects of water, sanitation, handwashing and nutritional interventions on soil-transmitted helminth infections in young children: A cluster-randomized controlled trial in rural Bangladesh
Source: PLoS Negl Trop Dis. 2019 May 3;13(5):e0007323. doi: 10.1371/journal.pntd.0007323 (PMC6519840; doi:10.1371/journal.pntd.0007323)
Supplement: S1 Table — (PDF) [file pntd.0007323.s007.pdf]

Table S1: Enrollment characteristics of individuals with missing vs. observed outcomes

No. of individuals	Missing (N=2824)	Observed (N=7187)
Index child, %	49.3	53.3
**Maternal**		
Age, mean	23.1	24.3
Years of education, mean	5.9	5.7
**Paternal**		
Years of education, mean	4.9	4.7
Works in agriculture, %	28.0	33.1
**Household**		
Number of persons, mean	4.6	4.8
Has electricity, %	56.0	59.3
Has a cement floor, %	12.2	9.6
Acres of agricultural land owned, mean	0.1	0.1
**Drinking water**		
Shallow tubewell primary water source, %	72.0	74.0
Stored water observed at home, %	50.6	46.7
Reported treating water yesterday, %	0.0	0.2
**Sanitation**		
Daily defecating in the open, %		
Adult men	7.6	8.3
Adult women	3.6	4.9
Children: 8-<15 years	9.1	11.0
Children: 3-<8 years	36.7	38.0
Children: 0-<3 years	86.2	83.7
Latrine, %		
Owned	51.7	51.5
Concete slab	94.2	93.4
Functional water seal	30.5	28.6
Visible stool on slab or floor	49.3	48.2
Owned a potty, %	5.7	4.5
Human feces observed in, %		
House	7.8	9.3
Childs's play area	1.3	1.2
**Handwashing**		
Has within 6 steps of latrine, %		
Has water	14.0	11.0
Has soap	8.6	5.6
Has within 6 steps of kitchen, %		
Has water	9.1	8.2
Has soap	2.4	2.5
