# Supplementary material for: Effects of water, sanitation, handwashing and nutritional interventions on soil-transmitted helminth infections in young children: A cluster-randomized controlled trial in rural Bangladesh
Source: PLoS Negl Trop Dis. 2019 May 3;13(5):e0007323. doi: 10.1371/journal.pntd.0007323 (PMC6519840; doi:10.1371/journal.pntd.0007323)
Supplement: S10 Table — (PDF) [file pntd.0007323.s016.pdf]

Table S10: Fecal egg count reduction, combined nutrition plus WSH vs. WSH and nutrition interventions

		Geo-	Geometric FECR [a]			Arithmetic FECR [a]		
Arm	N	mean	Unadjusted	Adjusted [b]	IPCW [c]	Unadjusted	Adjusted [b]	IPCW [c]
**Ascaris**								
Nutrition + WSH	933	5.1						
WSH	941	4.4	0.14 (-0.21, 0.49)	0.21 (-0.14, 0.55)	0.18 (-0.16, 0.53)	1.09 (-0.50, 2.68)	1.35 (-0.41, 3.12)	1.33 (-0.48, 3.14)
Nutrition	863	6.3	-0.16 (-0.41, 0.10)	-0.18 (-0.42, 0.07)	-0.17 (-0.42, 0.08)	0.42 (-0.60, 1.45)	0.41 (-0.59, 1.41)	0.43 (-0.57, 1.42)
**Hookworm**								
Nutrition + WSH	933	0.3						
WSH	941	0.4	-0.03 (-0.14, 0.07)	-0.03 (-0.13, 0.07)	-0.03 (-0.13, 0.08)	-0.52 (-0.81, -0.23)	-0.51 (-0.81, -0.22)	-0.51 (-0.81, -0.22)
Nutrition	863	0.6	-0.15 (-0.27, -0.04)	-0.14 (-0.26, -0.03)	-0.15 (-0.26, -0.04)	-0.72 (-0.94, -0.51)	-0.72 (-0.94, -0.51)	-0.72 (-0.93, -0.52)
**Trichuris**								
Nutrition + WSH	933	0.6						
WSH	941	0.4	0.14 (-0.06, 0.34)	0.16 (-0.03, 0.35)	0.14 (-0.05, 0.34)	0.48 (-1.01, 1.96)	0.48 (-0.98, 1.94)	0.46 (-0.93, 1.85)
Nutrition	863	0.4	0.13 (-0.06, 0.31)	0.13 (-0.04, 0.30)	0.13 (-0.04, 0.29)	1.46 (-0.53, 3.45)	1.42 (-0.50, 3.35)	1.45 (-0.76, 3.65)

[a] Faecal egg count reduction defined as egg ratio (ER) - 1, where ER is the ratio of mean eggs per gram between arms.

[b] Adjustment covariates considered include ID of the lab staff member who performed the Kato-Katz analysis, month of measurement, child age, sex and birthorder, mother's age, height and education, household food insecurity, number of children <18 years in household, number of individuals in compound, distance to the household's drinking water source, housing materials and assets. The adjusted model for each outcome includes covariates associated with the outcome at p<0.2 level in bivariate analysis.

[c] Inverse probability of censoring weighting. Adjustment covariates considered include the variables above except for ID of the lab staff member who performed the Kato-Katz analysis, month of measurement, child age, sex and birth order since this information is not available for individuals lost to follow-up. An indicator variable distinguishing index vs. non-index child status was included as a proxy for age.
